# Large-area 3D reconstruction of corneal tissues from oscillating focus confocal microscopy

**DOI:** 10.1038/s41598-026-48735-5

**Published:** 2026-04-17

**Authors:** Stephan Allgeier, Sebastian Bohn, Ralf Mikut, Klaus-Martin Reichert, Oliver Stachs, Elisabeth Wagner, Uwe Klaus Zettl, Karsten Sperlich

**Affiliations:** 1 Institute for Automation and Applied Informatics, Karlsruhe Institute of Technology (KIT), P.O. Box 3640, Karlsruhe, 76021 Germany; 2 Department of Ophthalmology, Rostock University Medical Center, Rostock, Germany; 3 Department Life, Light & Matter, University of Rostock, Rostock, Germany; 4 Department of Neurology, Neuroimmunology Section, Rostock University Medical Center , Rostock, Germany

**Keywords:** Cornea, In vivo confocal microscopy, Volume reconstruction, Volume registration, Subbasal nerve plexus, Dendritic cells, Biomedical engineering, 3-D reconstruction, Software, Biomarkers

## Abstract

In vivo confocal microscopy (IVCM) is being used extensively to examine the tissues of the human cornea at the cellular level. IVCM facilitates the examination of alterations of cellular features in a large variety of ocular and systemic pathologies that affect the cornea. A research focus has been on the corneal subbasal nerve plexus (SNP), but recent literature indicates a growing interest in IVCM examinations of immune cells in the cornea. Such applications are currently limited to the assessment of either single IVCM images, wide-field 2D representations of a laterally extended area (primarily of the SNP), or volume reconstructions from a single focus scan, but with a lateral extent of only a single field of view (for example for thickness measurements of corneal layers). The present contribution describes for the first time a workflow to generate large-area volume representations of corneal tissues from laterally extended 3D-IVCM datasets acquired with oscillating focus scans. Three novel techniques for volume reconstruction with different degrees of computational complexity are described, that decompose the dataset into separate focus stacks and create single-stack volume representations that are finally merged. The proposed methods differ in their capability to compensate axial movement occurring during the imaging process. The three novel methods are evaluated on 15 datasets of the SNP and the adjacent tissue layers. All three methods work well by visual inspection for the examined use case, but quantitative image quality assessment shows statistically significant improvement of the merged volume quality with axial motion correction techniques.

**Supplementary Information:**

The online version contains supplementary material available at 10.1038/s41598-026-48735-5.

## Introduction

In vivo confocal microscopy (IVCM) is being used extensively to examine the tissues of the human cornea at the cellular level. In particular, the high lateral and axial resolution that is achieved with laser-based devices has established confocal laser scanning microscopy (CLSM) as the de facto standard for IVCM examinations of the cornea. The majority of such examinations focus on the corneal subbasal nerve plexus (SNP), a dense network of nerve fibers between Bowman’s layer and the basal corneal epithelial cells. The SNP is constituted by the distal parts of axons of the ophthalmic branch of the trigeminal nerve^1,2^. Numerous studies have been conducted to characterize the healthy SNP^3,4^, examine characteristic alterations in various diseases such as diabetic neuropathy^5^, multiple sclerosis^6^, familial amyloid polyneuropathy^7^, Parkinson’s disease^8^ and long COVID^9^, or monitor changes in SNP morphology after therapeutic intervention or corneal surgery^10–13^.

Even though the monitoring of SNP changes is most often performed on long time scales with months or years in between examinations, the non-invasive nature of IVCM also allows repeated imaging sessions on much shorter time intervals such as weeks, days, or even within a single day. This has been used to describe and quantify the dynamic nature of the SNP^14,15^. Whereas SNP morphology can be analyzed either in single IVCM images or in wide-field montages of the SNP, the assessment of nerve migration requires wide-field montages or mosaics. Early SNP mosaics had been assembled manually^14,16,17^, but several automated approaches have been described since to make both the imaging process as well as the mosaicking process more efficient^18–25^.

In a recent study, Stoddard-Bennett et al. have compared the morphological characterization of the SNP in 2D vs. 3D image data in limbal stem cell deficiency. They show that the 3D analysis has advantages for some morphological features. In particular, the branching patterns of the SNP can be characterized more accurately in 3D representations^26^. Their 3D reconstruction was limited to single focus scans, though, and the authors state the extension of their analyses to wide-field 3D SNP image data as a desirable future research objective.

Recent literature also indicates a growing interest in IVCM examinations of corneal immune cells (IC). While the cornea is commonly described as an immune-privileged tissue, it contains a diverse population of resident ICs, including dendritic cells (DC), macrophages, innate lymphoid cells^27^, and T-cells^28^, which are distributed across both the epithelial and stromal layers.

The antigen-presenting DCs^29,30^ are predominantly found in proximity to the SNP, and play an important role in the maintenance of corneal nerves^31^. Bohn et al. have found a strong increase and a following rapid decrease of IC density that was closely related to the period of intake and the end, respectively, of a specific chemotherapy regimen^32^.

Interestingly, recent IVCM findings have shown that ICs are not static in the corneal tissue but that they can change their appearance and position within minutes^33,34^. For such IC movement analyses, multiple images have to be aligned and then (potentially) facilitate tracking individual ICs from image to image. Even though these first findings have been realized within the field of view of single IVCM images, a comprehensive examination to track ICs must be conducted over a larger area in order to be able to track the IC motion reliably. Another aspect to consider in this approach to the analysis of IC motion with CLSM is that the image plane of IVCM devices is perpendicular to the optical axis of the microscope. Therefore, a set of IVCM images (single images or large-scale mosaic montages) from the same tissue depth, most commonly the SNP level, at different time points only provides information on the lateral IC movement components within this image plane. Any potential depth component of the movement is not discernible in these data. If an individual IC moves to an adjacent tissue level, it can no longer be tracked, even if it reappears at the initial tissue level in a later image. Here and throughout the manuscript, the term “lateral” denotes any direction that is parallel to the image plane (as opposed to the more specific anatomical definition). It is further assumed that the image plane is generally parallel to the ocular surface. The coordinates x and y denote the two lateral spatial dimensions, the coordinate z is used for the remaining depth or axial component, i.e., the direction parallel to the optical axis of the IVCM device.

Therefore, in order to track IC movement along the depth dimension, it is necessary to record volume data. Existing volume reconstruction methods for corneal confocal microscopy only handle datasets that contain a single focus scan, often with a large scan depth extending over the majority or the entirety of the corneal thickness^26,35–37^. This is not viable for IC tracking because finding and imaging the same corneal location with respect to the lateral position is not feasible with volume sections that only have a lateral extent of a single field of view. Furthermore, even if the same area of this extent could be imaged repeatedly over time, this approach would limit the observable migration processes to only those that take place inside the confined lateral extent of the imaged volume.

Overall, the analysis and monitoring of the SNP’s 3D configuration and the task of tracking ICs over different periods of time both share similar requirements that are currently unmet. To address these processes in a meaningful and practicable way, it is essential that the region imaged at each imaging session possesses sufficient extent in all three dimensions. It requires a sufficient scan depth in order to fully contain the depth variability of subbasal nerves^26,38,39^ or to capture potential depth movement components of the ICs. At the same time, it needs enough lateral extent to (a) provide sufficient area for reliable SNP characterization or to capture the lateral movement of a sufficient number of ICs and (b) facilitate the alignment of several successive datasets for monitoring purposes or analysis of dynamic processes^15,40^.

**Fig. 1:**
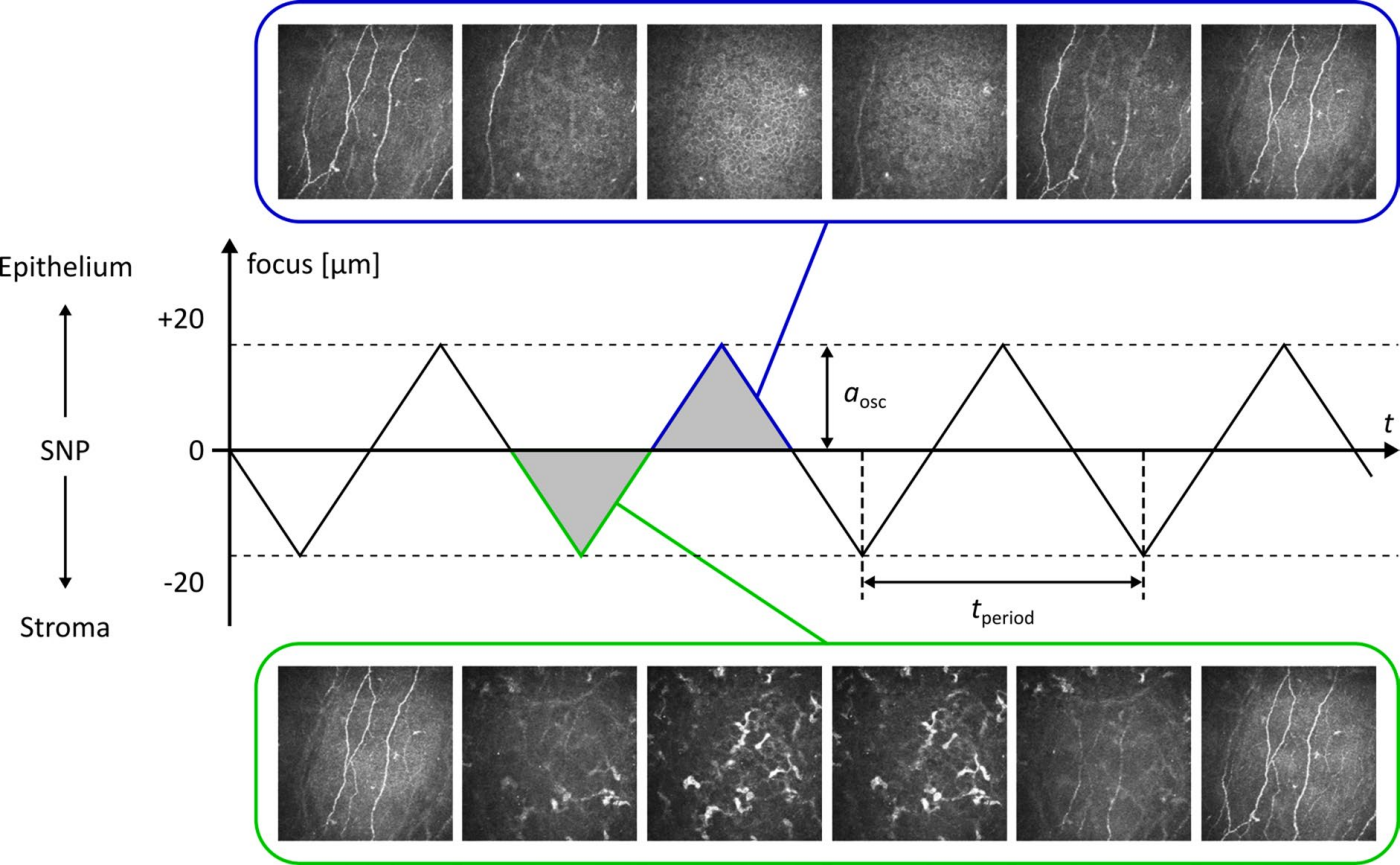
Oscillation of the focus plane during image acquisition. The focus depth variation describes a periodic triangular function. (Reproduced from^38^).

Such an imaging process has, in fact, been developed and described previously^38,41^ and has been in use for several years for the purpose of large-area imaging of the SNP^15,32,40,42^. For the lateral expansion of the imaged region, the described setup uses a moving fixation target in front of the non-examined eye to induce guided eye movements (in both eyes). An accompanying continuous oscillation of the focus depth provides the required axial extent of the recorded tissue region. For the use case of SNP imaging, the focus oscillation has an amplitude of ± 20 μm above and below the central SNP level so that adjacent tissue is regularly imaged in addition to the SNP structures (see Fig. 1). For the purpose of SNP imaging, the rationale for this procedure has been to make sure that the SNP layer is reliably recorded at every lateral position of the imaged corneal area, even in case of small variations of the depth of the SNP below the corneal surface. The non-SNP tissue images have been excluded during the image montaging process. Even though the IVCM datasets recorded in this manner contain image information from different tissue layers (covering an axial extent of 40 μm), the established post-processing workflow does not generate volume representations of the acquired tissue, but only 2D projections of the SNP.

However, the described process for large-area IVCM imaging can be modified to serve volumetric examinations such as 3D SNP analysis or IC tracking. In fact, the imaging process itself can be employed without modification, or with simple adjustments to the focus oscillation parameters such as the central tissue level, focus oscillation amplitude or focus scan velocity. The recorded data then needs to be post-processed in order to generate a volume representation of the imaged tissue. Neither the published large-area SNP montaging techniques^18–25^ nor the mentioned volume reconstruction methods^35–37^ are capable of this.

The present contribution presents a solution that combines the capabilities of large-area lateral montaging with volume reconstruction. We describe a complete post-processing pipeline to reconstruct a volume representation of the entire imaged area for datasets recorded as described above. Because the novel post-processing methods do not affect the imaging part of the entire workflow, they can also be applied to existing datasets. We provide a detailed description of the methods involved and present the results of the volume reconstruction for exemplary datasets of the SNP and the adjacent tissues.

## Methods

**Fig. 2:**
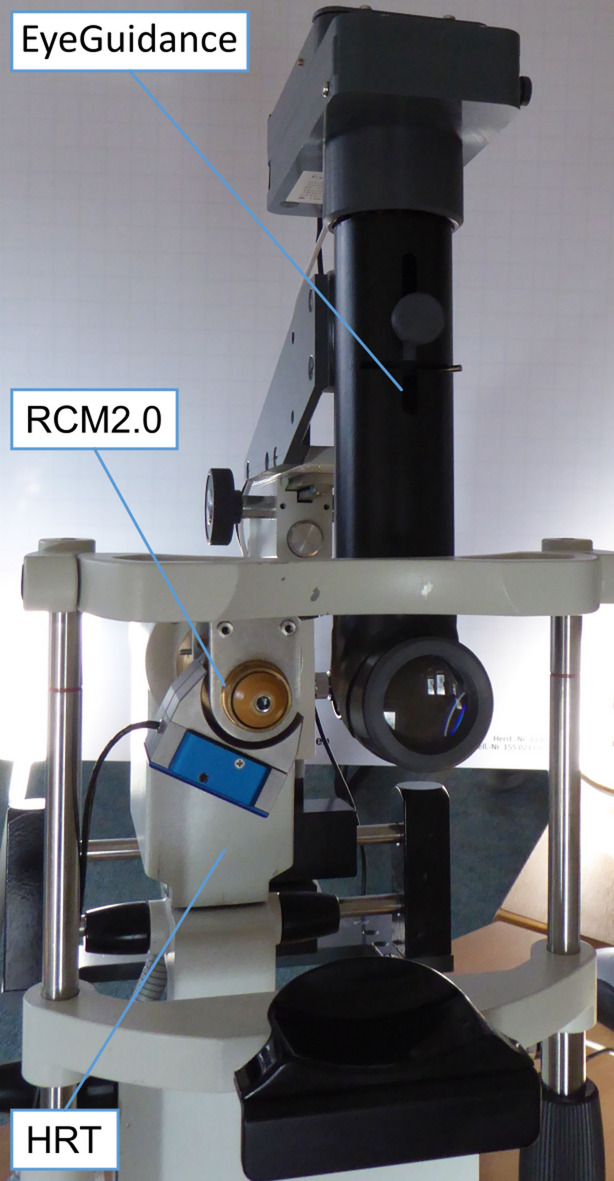
The imaging setup combines an HRT3 confocal microscope with the RCM 2.0 for fast and precise focus control and the EyeGuidance system to induce voluntary eye movements for large-area imaging. (Adapted from^41^).

The imaging process used to record the raw image datasets has been described in full detail earlier^38^. It builds on and extends the established HRT-RCM platform (Heidelberg Retina Tomograph and Rostock Cornea Module, both Heidelberg Engineering, Heidelberg, Germany). Specifically, it uses an HRT3 (Heidelberg Engineering, Heidelberg, Germany), the EyeGuidance system^23^ and the RCM2.0^37^ as shown in Fig. 2. The EyeGuidance system is a research prototype device developed at KIT (Karlsruhe, Germany) that serves to guide the view direction of the examined participant during imaging in order to record a laterally extended area of the cornea. The RCM2.0 is a research prototype developed at the Rostock University Medical Center (Rostock, Germany). It replaces the commercial RCM and provides precise high-speed focus control, which is used to create a continuous oscillation of the focus plane in a triangular waveform throughout the imaging process. The current focus plane position is obtained from the integrated actuator’s closed-loop position sensor and stored separately. In effect, the recorded raw HRT image sequence (384 × 384 pixels, 350 × 350 μm², 0.9 × 0.9 μm² pixel size, 30 frames per second) forms a series of focus scans that alternate between an anterior and posterior focus scan direction.

**Fig. 3:**
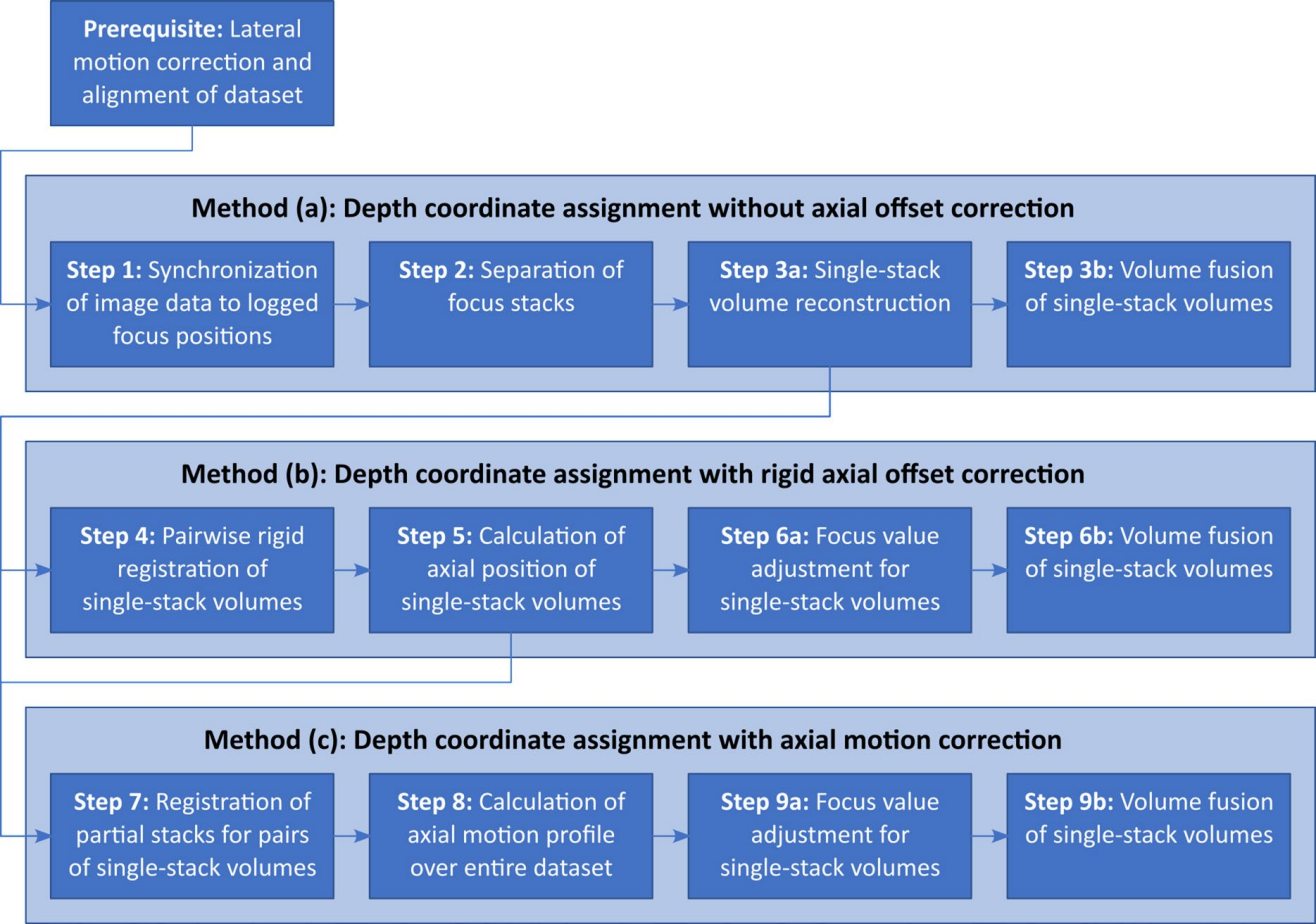
Overview of the process pipeline. The three methods directly build on top of each other as the result of some steps of methods (a) and (b) are required as the base input of methods (b) and (c), respectively. Surrounding boxes highlight the separation of the three different methods.

We propose three novel approaches that build on top of each other and possess successively increasing processing complexity (along with successively increasing processing time). Figure 3 provides an overview of the distinct process steps of the complete pipeline; surrounding boxes mark the three approaches. All three methods depend on image datasets that have already been corrected for lateral motion components (i.e., motion artefacts that manifest in the IVCM image plane) and are aligned with respect to their lateral coordinates, as described in the following section. What remains is to determine the $\:z$-coordinate associated with each image.

The three approaches to be compared are:

- Method (a): Volume reconstruction based on recorded image scan depths (i.e., without axial offset correction), as described in the following sections.
- Method (b): Volume reconstruction with rigid depth registration (i.e., with rigid axial offset correction), as described in the following sections.
- Method (c): Volume reconstruction with non-rigid depth registration (i.e., with axial motion correction), as described in the following sections.

In principle, the contact of the eye with the TomoCap (a single-use contact element that is mounted on the RCM2.0 and used as a contact element during the imaging process) essentially prevents large motion components in the axial direction. However, if the contact with the eye is kept minimal, then small movements in the axial dimension are possible. In addition, the corneal tissue is not rigid, potentially allowing axial tissue compression to occur. From method (a) towards method (c), the capability to address these potential axial movement effects increases successively.

### Prerequisite: lateral motion correction and alignment of the dataset

Because of lateral rotational eye movements during the image acquisition process, the single images of a focus scan cannot simply be stacked to create a volume representation. The eye movements – both involuntary fixational eye movements and voluntarily induced eye movements, for example through the EyeGuidance^23,38^ system – lead to relative lateral translations and also induce characteristic geometric distortion artifacts in the image frames. These motion artifacts have to be corrected before the actual volume reconstruction can be obtained. The motion correction algorithms have been described in more detail^43,44^ as part of the SNP mosaic image generation process^38,41^. The motion-corrected version of all images in a dataset is calculated as an intermediate step in the mosaic image formation process, just before they are blended for the large-area 2D representation of the SNP. It is important to note here that the motion-corrected images are all represented in a common pixel grid (which is the mosaic image pixel grid for the mosaic image creation application).

### Method (a): depth coordinate assignment without axial offset correction

The underlying assumption for this approach is that no axial movement of the imaged tissue occurs during the imaging process, so axial position corrections are not required. The z-coordinates associated with the images are taken from the focus value log of the RCM2.0. The imaging process (controlled by the HRT software) and the focus oscillation (controlled by the RCM2.0 software) are only synchronized by a common software trigger to start simultaneously; apart from this, they run independently. Therefore, the focus values have to be associated with the image data using their separate time stamps.

#### Step 1: synchronization of the image data to the logged focus positions

Even though the image and focus depth data are recorded in two separate and independent software processes, both data streams carry time stamps. At first glance, assigning a focus depth value to each image (or sub-image region) seems to be a straightforward, trivial task. There are two reasons why this is not the case. First of all, the (single) time stamp of an image is assigned by the proprietary HRT control software, and it is unknown at which point of the imaging process the time associated with each image is taken. It could be the time the first pixel is imaged, the last pixel, or somewhere in between; it could even be at some unknown time after the image is completely recorded. The second reason is that the imaging process and the focus control process run on separate PCs, and exact absolute clock synchronization with the required accuracy (in the order of 1–2 milliseconds) is very challenging. Rather, we determine and log the offset between the two clocks by exchanging specific initial messages over the network interface during the initialization of the imaging process. An additional, experimentally determined time constant accounts for the unknown time point at which the time stamp is taken and for any other constant latency effects.

#### Step 2: separation of focus stacks

**Fig. 4:**
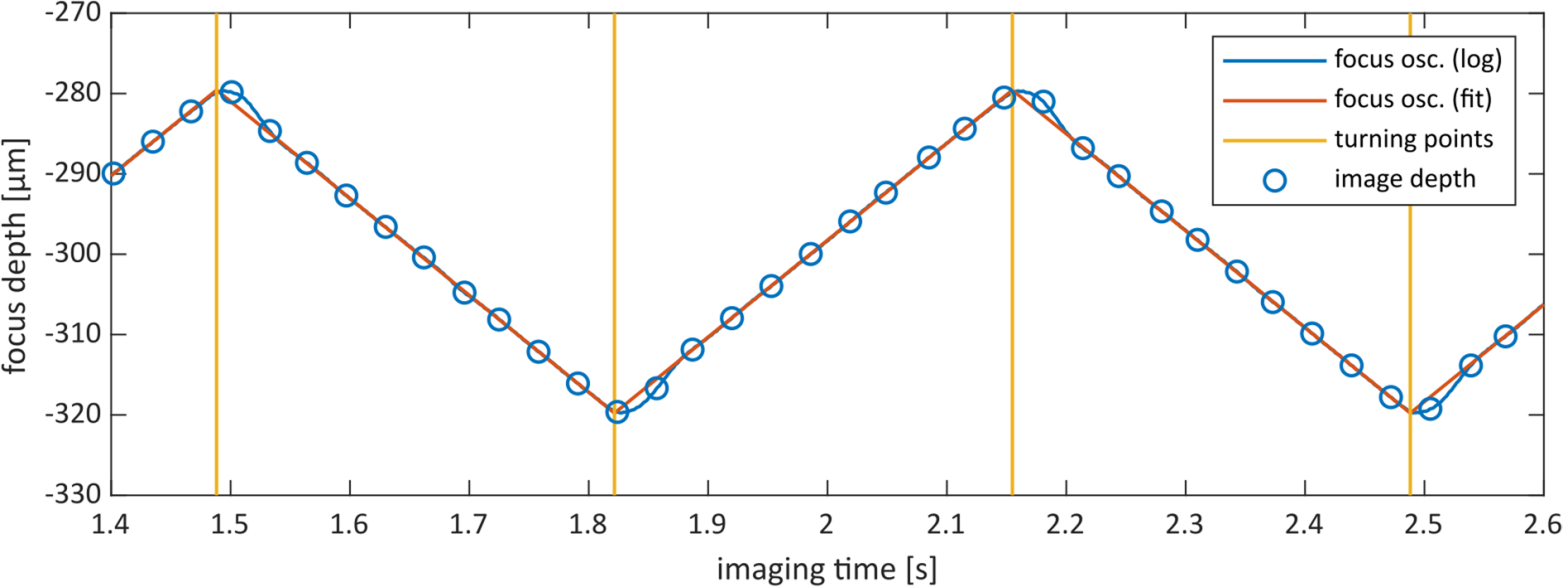
Dataset decomposition into separate focus stacks (step 2 of the workflow). The diagram shows a section of 1.2 s of the focus oscillation process of the RCM2.0 (blue), and the ideal triangular signal (orange) that is fitted to the logged focus data. The focus turning points (yellow) are estimated from the fitted triangular signal and define the decomposition of the dataset into focus stacks: all images in between two consecutive turning points constitute a separate, unidirectional focus stack. The depth coordinates associated with the image data (blue circles) are the result of the synchronization step 1.

For the subsequent volume reconstruction step, the dataset is decomposed into separate focus stacks, each containing exactly the images recorded in between two adjacent anterior and posterior focus oscillation turning points (see Fig. 4). The exact time points of the focus direction change are calculated simply by fitting an ideal triangular signal form to the logged focus values. Even though the focus turning points are what we are looking for, they are also the points that inevitably deviate from the ideal desired triangular waveform. To avoid systematic errors, only the focus values near the central focus level are used for the fitting process; the top 25% and the bottom 25% focus values are excluded. Each stack represents a single continuous unidirectional focus scan.

#### Step 3a: single-stack volume reconstruction

In order to obtain a voxel volume representation of the imaged 3D area, we need to specify the regular voxel grid and then assign an intensity value to every voxel. The $\:x$- and $\:y$-components of the voxel grid are already established by the common pixel grid of the motion-corrected images. For the $\:z$-component of the voxel grid, we start by defining the central depth value $\:{z}_{\mathrm{c}\mathrm{e}\mathrm{n}\mathrm{t}\mathrm{e}\mathrm{r}}$ of the focus oscillation process as a reference level of the voxel grid. We further define the constant distance $\:{z}_{\mathrm{s}\mathrm{t}\mathrm{e}\mathrm{p}}$ between adjacent voxel grid positions along the z-axis (in other words, the voxel height) to be equal to the pixel dimension, i.e., $\:{z}_{\mathrm{s}\mathrm{t}\mathrm{e}\mathrm{p}}$ = 0.9 μm. In principle, the voxel height can be chosen arbitrarily. The decision for a cubic voxel grid creates an isotropic representation of the imaged tissue and is particularly useful for geometric measurements in the reconstructed volume. Two reasonable alternative choices for $\:{z}_{step}$ would be the axial optical resolution of the imaging system (4 μm^45^) or the mean distance between successive HRT image frames (3.9 μm for our imaging protocol). The voxel grid then simply extends above and below the reference level as far as needed to contain all image depth values. We can now create single-stack volume representations from each focus stack. Because the image depth values are generally not at one of the voxel grid $\:z$-levels, we need to interpolate the voxel intensities at the voxel grid positions; we use a simple linear interpolation of the two adjacent motion-corrected images above and below the grid level. No extrapolation is performed beyond the last recorded image at both ends, so the top- and bottom-most voxel grid levels may remain empty for several focus stacks. This needs to be considered when specifying the focus oscillation parameters, if a minimal axial extent of the generated single-stack volumes is required.

#### Step 3b: volume fusion of the single-stack volumes

Finally, the single-stack volume images are merged into one volume representation by simple averaging. All single-stack volumes are represented in the same, common voxel grid; hence, no further interpolation is required. The averaging is implemented as calculating the mean value from all single-stack volumes at each voxel grid position. For a given voxel position, only single-stack volumes that contain information in this voxel are considered for this averaging process.

### Method (b): depth coordinate assignment with rigid axial offset correction

The underlying assumption for this approach is that no (or only negligible) axial movement occurs during the acquisition of a single focus stack. Each stack is regarded as rigid. However, axial offsets between stacks might occur and need to be corrected. The adjustment value and the resulting axial position for each stack are determined using a rigid image registration technique. As shown in Fig. 3, the process steps of this method build upon steps 1 through 3a from the previous section, using the reconstructed volume representations of the single focus stacks as input data.

#### Step 4: pairwise rigid registration of the single-stack volumes

In analogy to the 2D image registration process used for the lateral motion correction and alignment of the raw image sequence, the 3D phase correlation function is used to determine a 3D translation vector to align a pair of single-stack volumes. The correlation function’s maximum specifies the relative offset of the two stacks. Since all stacks are already aligned in both lateral dimensions (because they have been composed of laterally motion-corrected and aligned images), the x- and y-components of the offset vector should both be very close to zero. This fact can be used effectively to validate the phase correlation result: if the lateral offset deviates from zero by more than a low threshold (we use two pixels in both dimensions), then the correlation result is known to be incorrect. This may occur when the overlapping area of the two volumes is small or only contains low-contrast image information, if the two stacks are in fact inconsistent because of an artifact (of arbitrary origin) in either of the stacks, or theoretically if they do not overlap because of an axial offset value larger than the stack height (stack pairs that nominally don’t overlap according to their lateral alignment are not considered for registration). In the opposite case, if the calculated lateral offset is small enough, we assume the correlation result to be correct. All accepted registration results are collected in a list noting the stack indexes $\:i$ and $\:j$ and the relative axial offset value $\:d{z}_{i,j}$.

#### Step 5: calculation of the axial position of the volume stacks

Each of the accepted axial offset values $\:d{z}_{i,j}$ defines the assumed difference between the unknown absolute axial positions $\:{z}_{i}$ and $\:{z}_{j}$ of the volume pair, $\:\:d{z}_{i,j}={z}_{j}-{z}_{i}$. Together, these equations form a simple system of linear equations with the absolute axial positions of all single-stack volumes as the unknowns. By including a regularization constraint, e.g. defining the axial position of the first stack $\:{z}_{1}=0$, we can solve the system for the absolute axial positions. In some cases, the axial position correction exhibits a global tilt, where stacks located towards a certain $\:x$-$\:y$-direction are shifted upwards and stacks located diametrically opposed are shifted downwards. To compensate for this, the best-fitting plane is calculated using the known $\:x$- and $\:y$-coordinates and the calculated axial positions of the stacks. For each stack, the $\:z$-value of the best-fitting plane at the lateral position of the stack is then subtracted from its previously calculated axial position value.

#### Step 6a: focus value adjustment for the single-stack volumes

The volume reconstruction is now rather simple. The focus depth associated with each image is adjusted according to the absolute position value of the single-stack volume in which it is included; the single-stack volume reconstruction process is then repeated as described in step 3a.

#### Step 6b: volume fusion of the single-stack volumes

Using the new single-stack volumes, the volume fusion process is then identical to step 3b.

### Method (c): depth coordinate assignment with axial motion correction

The underlying assumption of this approach is that axial movement occurs throughout the imaging process and that these movements are smooth, i.e., with limited acceleration. Such movement would induce geometric distortions in the volume data, namely axial stretching or compression of the volume, depending on the tissue’s axial movement direction relative to the focus scan. The axial movement over time is deduced from the image data and subsequently corrected. To some degree, the process is analogous to the lateral 2D registration process of the raw HRT image data. As shown in Fig. 3, the process steps of this method build upon steps 1 through 6a from the previous sections, using the reconstructed single-stack volume representations from step 3a and additionally their absolute axial positions from step 5 as input data.

#### Step 7: registration of partial stacks for pairs of single-stack volumes

**Fig. 5:**
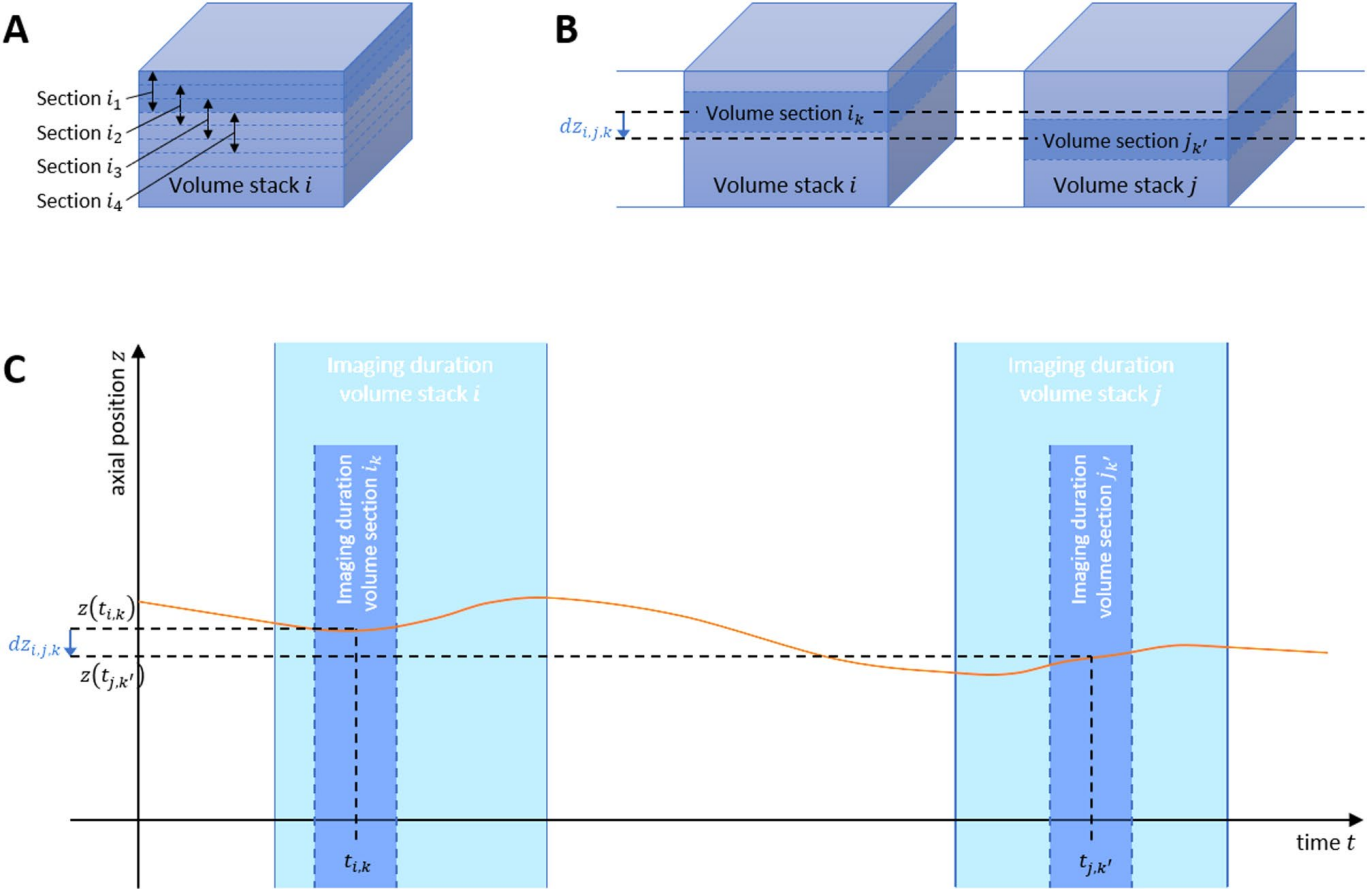
Schematic visualization of steps 7 and 8 of method (c). (**A**) Decomposition of a single-stack volume into successive partial stacks. (**B**) Two volume representations $\:i$ and $\:j$ and two corresponding partial stacks (partial stack $\:{i}_{k}$ of volume $\:i$ and partial stack $\:{j}_{{k}^{{\prime\:}}}$ of volume $\:j$) and the registration result, that suggests a relative axial offset value of $\:d{z}_{i,j,k}$ between both partial stacks. (**C**) Visualization of the (unknown) axial movement trajectory $\:z\left(t\right)$ (orange) and the imaging periods of the stacks and partial stacks from subfigure (**B**) as well as the time points $\:{t}_{i,k}$ and $\:{t}_{j,{k}^{{\prime\:}}}$ that are associated with the shown corresponding partial stacks. The measured relative axial offset value $\:d{z}_{i,j,k}$ equals the difference $\:z\left({t}_{j,{k}^{{\prime\:}}}\right)-z\left({t}_{i,k}\right)$.

As with step 4 above, this procedure is applied to each possible combination of two distinct single-stack volumes. For each pair of single-stack volumes, one of the stacks is decomposed into a set of partial stacks, as shown in Fig. 5A. Basically, a partial stack with a height of 12 voxels is cropped from the volume at each axial integer coordinate of the voxel grid. Each partial stack represents the image data acquired during a specific continuous time interval of the imaging process. Based on the position estimation of both stacks from step 5, each partial stack is then registered (rigidly) with the estimated corresponding partial stack of the other single-stack volume, again using the 3D phase correlation function. As described in step 4, the position of the maximum of the phase correlation function specifies a 3D translation vector that describes the offset between both partial stacks. The $\:z$-component of that vector specifies the relative axial offset $\:dz$ of the registered partial stacks. The x- and y-coordinates of the maximum are again used to validate the registration result, as described in step 4. Because of the choice of the pair of partial stacks based on the pre-positioned volumes, $\:dz$ is to be interpreted as an adjustment value for the estimation of the relative offset between the two partial stacks. If the axial adjustment value is larger than half a voxel, the partial stack registration is repeated iteratively with an adjusted estimation of the corresponding partial stack. The iteration continues until the adjustment value converges or until the number of iterations surpasses a specified maximum number. If the process converges, two corresponding partial stacks have been found, namely partial stack $\:{i}_{k}$ of volume $\:i$ and partial stack $\:{j}_{{k}^{{\prime\:}}}$ of volume $\:j$ as shown in Fig. 5B. The final, adjusted relative offset between the two partial stacks is termed $\:d{z}_{i,j,k}$.

#### Step 8: calculation of an axial motion profile over an entire dataset

As before in step 5, the partial stack registration results describe relative axial offsets (i.e., position differences) between specific parts of the dataset. In effect, each partial stack is associated with the (known) interval along the time axis during which its image data was recorded (see Fig. 5C). Each determined axial offset value $\:d{z}_{i,j,k}$ between partial stack $\:{i}_{k}$ of volume $\:i$ and partial stack $\:{j}_{{k}^{{\prime\:}}}$ of volume $\:j$ therefore describes the position change that has occurred in the intermediate time after the first partial stack and before the second partial stack had been imaged. We now collapse the time interval associated with a partial stack to its central time point, so each partial stack corresponds to exactly one known point on the time axis. If $\:{t}_{i,k}$ and $\:{t}_{j,{k}^{{\prime\:}}}$ are the time points associated with partial stacks $\:{i}_{k}$ and $\:{j}_{{k}^{{\prime\:}}}$, respectively, then $\:d{z}_{i,j,k}$ equals the difference $\:z\left({t}_{j,{k}^{{\prime\:}}}\right)-z\left({t}_{i,k}\right)$ of the unknown absolute axial position coordinates at the respective time points. In total, we effectively have a large collection of relations between pairs of points along the (unknown) axial motion trajectory of the imaged tissue. Similar to step 5, these relations again form a system of linear equations that can be solved for the unknown absolute axial position values along the trajectory $\:z\left(t\right)$. In analogy to the 2D motion-correction process^43^, we need a regularization constraint here, in addition to defining the start of the trajectory to be at position zero. The regularization term forces the trajectory to be smooth, as mentioned above. It is essentially the second derivative, which is minimized together with the residual of the equation system. As with method (b), the resulting axial position trajectory $\:z\left(t\right)$ can exhibit a global tilt behavior that correlates with the lateral position (see Fig. 5). This is compensated again by calculating the best-fitting plane and then correcting the trajectory values by subtracting the $\:z$-values of the plane at the corresponding $\:x$-$\:y$-position.

#### Step 9a: focus value adjustment for the single-stack volumes

The volume reconstruction process is almost the same as described in step 6a. The only difference is that instead of applying the same focus depth adjustment to all images of a single focus stack, the focus depth adjustment for each image is now directly calculated from the axial motion trajectory, taking the trajectory value at the (known) acquisition time of the respective image.

#### Step 9b: volume fusion of the single-stack volumes

Using the new single-stack volumes, the volume fusion process is then identical to step 3b.

### Data description

For the experimental evaluation and comparison of the proposed approaches, we have applied the methods to 15 datasets acquired from 15 persons (8 female, 7 male; age distribution (mean ± SD): 44.9 ± 13.5 years) diagnosed with different forms of dry eye disease. Among other examinations, the patients underwent confocal microscopy to image the SNP. The datasets have been recorded with a focus oscillation peak-to-peak amplitude of 40 μm and a focus shift speed of 120 μm/s; however, the volume reconstruction process does not rely on these specific values. Furthermore, the exemplary datasets were recorded using the EyeGuidance system. However, the EyeGuidance system is not a prerequisite for the described method. The typical imaging time for a single dataset with the used imaging protocol is 40 s, which results in datasets of approximately 1200 raw IVCM images. With the chosen focus oscillation parameters and a frame rate of 30 frames per second, each unidirectional focus scan consists of approximately 10 raw images after the stack decomposition step, resulting in approximately 120 separate focus stacks per dataset.

The study from which the exemplary datasets were taken was approved by the Institutional Review Board of the Rostock University Medical Center [Rostock University Medical Center Ethics Committee, Registration number: A 2023 − 0158 (dated 20 September 2023)]. Informed consent was obtained from all participants. All examinations and experiments were conducted in accordance with the applicable laws, rules, and regulations of the Rostock University Medical Center.

### Comparison of the three methods

The three methods are compared visually based on the merged volume representations of the recorded tissue regions after steps 3b, 6b and 9b. In particular, transversal and sagittal depth section images (parallel to the x-z- and y-z-planes, respectively) through the reconstructed volumes are assessed for a qualitative comparison.

For quantitative comparison, the ARDE (automated reference detection estimator) metric^46^ is calculated for frontal, transversal and sagittal section images. That metric is an NR-IQA technique (no-reference image quality assessment) that has been developed specifically for CLSM images. First, the mean ARDE values for frontal, sagittal and transversal images are calculated for each dataset and method individually. To detect significant differences between the methods, the Friedman test is applied, as the residuals of the data sets are not normally distributed (Shapiro-Wilk, α = 0.05) and there is no sphericity (Mauchly test, α = 0.05). For pairwise comparisons of the three methods, the Wilcoxon signed-rank test (α = 0.05) with Bonferroni correction is used. All statistical analyses are made using IBM SPSS Statistics (version 29.0.1.1, IBM Corp., Armonk, NY, USA).

Further conclusions are drawn from the estimated trajectories $\:z\left(t\right)$.

## Results

**Fig. 6:**
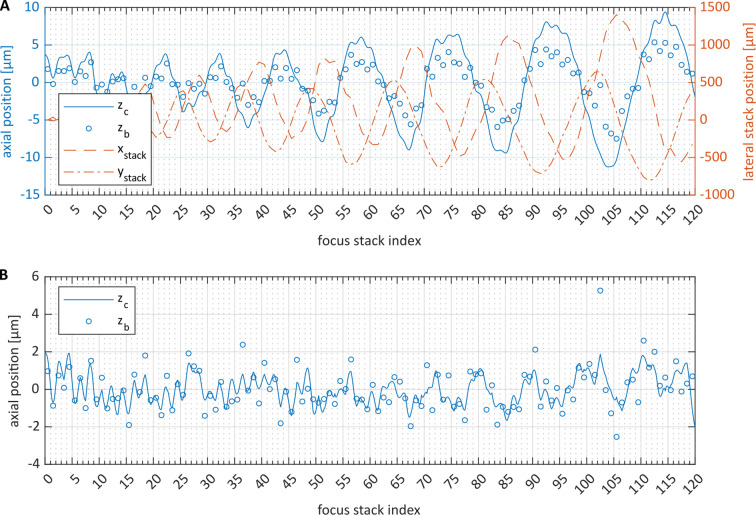
Axial position correction for a typical dataset. Small blue circles (labeled $\:{z}_{b}$) represent the axial stack positions calculated in step 5 of method (b); the continuous solid blue line (labeled $\:{z}_{c}$) plots the axial offset of the partial stacks calculated in step 8 of method (c). Subfigure (**A**) visualizes the axial offsets before the global tilt correction in steps 5 and 8; the dashed and dotted red lines plot the central x- and y-coordinates, $\:{x}_{\mathrm{s}\mathrm{t}\mathrm{a}\mathrm{c}\mathrm{k}}$ and $\:{y}_{\mathrm{s}\mathrm{t}\mathrm{a}\mathrm{c}\mathrm{k}}$, of each focus stack. Subfigure (**B**) visualizes the axial offsets after the global tilt correction in steps 5 and 8.

**Fig. 7:**
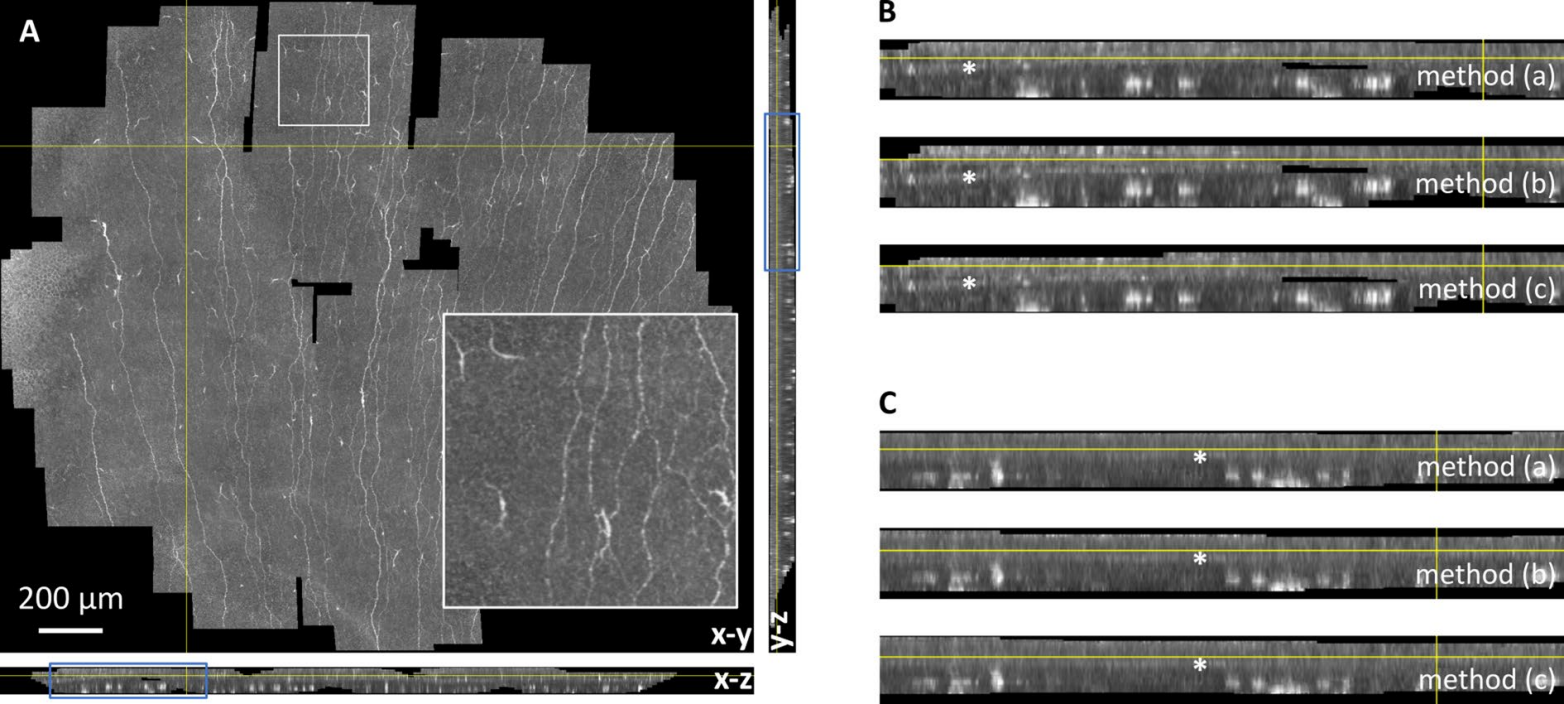
Sections through the reconstructed merged volume. (**A**) orthogonal section images parallel to the x-y-plane (frontal, large image), the x-z-plane (sagittal, below the frontal image), and the y-z-plane (transversal, to the right of the frontal image). The inset in the bottom right corner of the frontal view is a magnification of the white-bordered region at the top of the frontal view. (**B**) Magnified area of the transversal section (marked by a blue rectangle in (**A**)); from top to bottom: methods (a), (b) and (c). (**C**) Magnified area of the sagittal section (marked by a blue rectangle in (**A**)); from top to bottom: methods (a), (b) and (c). In subfigures (**B**) and (**C**), Bowman’s layer is visible as a horizontal layer that is slightly brighter than the adjacent epithelial and stromal tissue. Bowman’s layer is marked by asterisks, the epithelium is located above Bowman’s layer, the stroma is located below in these section views.

All 15 datasets were processed successfully with all three proposed novel methods. Results from a typical dataset are provided in Figs. 6 and 7. Figure 6 shows the calculated axial position adjustments for the single focus stacks, which are indexed along the horizontal axis. Both the stack adjustment values of method (b) (small circles) and the partial stack position adjustments of method (c) (continuous solid line) are shown. Figure 6A presents the calculated axial positions before the correction of the global tilt described in steps 5 and 8. The estimated axial positions (both with methods (b) and (c)) manifest an obvious oscillation phenomenon that increases both in amplitude and in period length over time. This oscillation correlates very well with the $\:x$- and $\:y$-coordinates of the stacks, i.e., with the spiraling movement of the eye under the microscope. Figure 6B shows the estimated axial position values of the same exemplary dataset after the global tilt has been compensated. The corrected trajectory reveals a remaining oscillating signal on a much smaller scale, with an amplitude in the order of 1–2 μm and a period of two stacks. Every second stack is shifted in the anterior direction and every other stack is shifted in the posterior direction.

Figure 7 provides orthogonal section images through the reconstructed merged volume representation of the same dataset. A part of the transversal and sagittal sections is shown in Fig. 7B,C to compare the results of methods (a), (b) and (c) (from top to bottom). The depicted depth sections reveal no qualitative differences between the three proposed methods. Again, this observation was made for all 15 examined datasets (see Suppl. Fig. S1-S15).

The results for the image quality assessment are given in Table 1. The quantitative comparison reveals a trend for small improvement of the result quality from method (a) to method (b) and from method (b) to method (c). Statistically significant differences were found between methods (a) and (c) for all section orientations, whereas significant differences between methods (a) and (b) were observed only for the frontal section images. No significant differences were found between methods (b) and (c).

**Table 1:** Qualitative and quantitative assessment of results. Quantitative results are given as median [first quartile; third quartile] of the ARDE values from the 15 processed datasets (first three rows) and as p-values for pairwise comparisons of all three methods (last three rows). Statistically significant differences are marked with bold text.

	Qualitative	Quantitative		
	Visual quality	Frontal	Sagittal	Transversal
Method (a)	good	10.21 [6.96; 12.06]	10.31 [7.73; 11.48]	10.63 [7.72; 12.71]
Method (b)	good	10.76 [7.34; 16.76]	10.34 [7.81; 13.10]	10.57 [7.81; 14.42]
Method (c)	good	11.29 [7.62; 17.20]	10.45 [7.93; 12.75]	10.66 [7.96; 14.11]
Comparison (a)-(b)	equal	***p*** **< 0.05**	*p* = 0.362	*p* = 0.166
Comparison (a)-(c)	equal	***p*** **< 0.0001**	***p*** **< 0.001**	***p*** **< 0.001**
Comparison (b)-(c)	equal	*p* = 0.166	*p* = 0.107	*p* = 0.166

The described procedures are implemented in MATLAB (version R2022b, The MathWorks Inc., Natick, MA, USA); the prerequisite lateral motion correction and alignment are implemented in C++. The datasets were processed on a workstation PC (OS: Microsoft Windows Server 2019; CPU: AMD Ryzen Threadripper PRO 3975WX; RAM: 256 GB). The processing times were 12.6 min for method (a), an additional 100.3 min for method (b) (93.9 min for step 4; 6.4 min for steps 5 and 6), and an additional 175.6 min for method (c) (156.1 min for step 7; 19.5 min for steps 8 and 9). Table 2 gives an overview of the measured processing times.

**Table 2:** Processing times and visual result quality. The processing times are listed as mean ± standard deviation from the 15 processed datasets; the processing time listed for method (b) includes the processing time for steps of method (a) that it builds on; the processing time listed for method (c) includes the processing time for steps of methods (a) and (b) that it builds on.

	Processing time (mean ± SD) [minutes]
Method (a)	12.6 ± 1.7 min
Method (b)	112.8 ± 15.4 min
Method (c)	288.4 ± 42.4 min

## Discussion

As with 2D mapping techniques or common methods for volume reconstruction from single volume scans or any other procedure that involves image data fusion, the accurate correction of potential motion artifacts is a fundamental requirement for any meaningful downstream application in which the image fusion result is being used. Accordingly, the prerequisite for the presented workflow is the correction of lateral motion artifacts, whether they originate from voluntary eye movements induced by the EyeGuidance system (or any other means to guide the view of the examined person) or from fixational eye movements^47^ when using a stationary fixation target or from other sources such as pathological nystagmus^48^. In contrast to the use cases of 2D mapping from IVCM data and volume reconstruction from single focus scans, the target application of the presented workflow, the fusion of volumetric data from several focus scans, would also require the correction of axial motion artifacts. Otherwise, non-corresponding image data might be averaged in the fusion process, with the consequence of reducing result quality.

As described earlier, large axial motion components are effectively reduced by the contact of the eye with the TomoCap during the imaging process. However, axial movement still occurs on a smaller scale, due to deformation of the corneal tissue, but also because minimizing contact pressure is generally recommendable in IVCM of the cornea to avoid the formation of tissue folds^49^. The purpose of the experiments with the proposed methods (b) and (c) was to examine whether there are notable axial motion artifacts in the data recorded with the described imaging workflow for SNP imaging^38^. The presented visual inspection results of 15 datasets suggest that this is not the case. The resulting visual quality with the simple depth coordinate assignment used in the proposed method (a) based on the logged focus values is good for all 15 datasets and does not increase when using the proposed axial registration methods. The quantitative image quality assessment using the ARDE metric comes to a different conclusion. Even though the relative quality metric improvement is small, it has been found to be statistically significant for all three section orientations between method (a) and method (c). This result is probably based on the increasing capability to correct axial motion artifacts from method (a) to method (b) and again to method (c). Whereas no such capability is present in method (a), method (b) can adjust the axial position of the separated focus stacks and therefore compensate slow, approximately linear shifts along the axial direction. However, the single focus stacks are considered rigid in method (b). Method (c) overcomes this limitation and is capable of correcting even axial motion effects inside the focus stacks by its partial stack registration approach. The concluding recommendation is to choose method (c) whenever the merged volumes are to be used for further algorithmic analyses and the required time resources are available.

The origin of high-frequency oscillations that are observed after correcting the global tilt effect is not entirely clear. The period of exactly two stacks (or 0.67 s) strongly suggests that this phenomenon does not describe actual tissue movement but is rather artificially generated. It might be attributable to the fact that the focus shift is oriented in opposite directions in every pair of consecutive focus stacks and therefore the actual image planes of the raw 2D images are skewed in opposite directions. However, the most probable explanation is that this points to an inaccurate time synchronization between image data and logged focus values. For an illustrative example, assume a time synchronization error of 4 ms. At a focus shift speed of 120 μm/s, the focus values assigned to each image would be 0.48 μm off. Therefore, if the images of a given focus stack are assigned z-coordinates that are 0.48 μm smaller than the actual recording depth, then the images of the following focus stack would be assigned z-coordinates that are 0.48 μm larger than the true recording depth. In order to overlap correctly, they would have to be corrected by almost 1 μm in the axial dimension relative to each other. This is exactly what we see in the resulting calculated axial correction plots. An improved image-focus-synchronization protocol to correct systematic time shifts may reduce this problem and running the focus control software on the same PC that also controls the HRT would remove it completely (apart from a constant time offset to compensate the unknown time point at which the image time stamp is generated, but this constant would have to be calibrated accurately only once). However, an erroneous relative axial offset of 1 μm is much smaller than the optical axial resolution of the imaging system of approx. 4 μm^45^. This could explain why the results of method (a), which are affected by this inaccuracy, are qualitatively equal to the results of methods (b) and (c), in which the inaccuracy is corrected.

The presented results that have been obtained from the examination of 3D SNP datasets may need to be revisited when looking at different use cases. For example, when imaging the superficial cell layer of the corneal epithelium it is essential to even have a small gap, which is bridged with contact gel, between the TomoCap and the ocular surface. Otherwise, the different refraction indices create a strong Fresnel reflection that entirely saturates the detector and inhibits the detection of any signal from the superficial tissue layer. Consequently, the cornea position is no longer axially rigidly fixed. When pursuing extended 3D imaging in this scenario, axial alignment correction of the image data can become necessary. An example for this use case is provided in Suppl. Fig. S16. When thinking even further and envisioning potential non-contact applications for IVCM imaging of the cornea, axial registration of 3D image data will certainly become necessary to compensate for relative axial movement components originating from breathing, pulsation or head movements.

Our main motivation for developing the presented method and the intended application of the created wide-field 3D reconstructions is the tracking of dendritic cell migrations in the vicinity of the SNP. However, there are other apparent use cases in which the technique could be immediately useful. One example is the characterization of the SNP in volumetric representations of the corneal tissue, as recently proposed by Stoddard-Bennett et al.^26^. In their study, they show the benefits of 3D SNP analysis, but they were limited to volumes with a lateral extent of a single field of view and state the extension of their analyses to wide-field 3D image data as a desirable future research objective. The methods presented in the current contribution could be used without any changes to this end. With parameter adjustments, the presented workflow could also be applied to image extended volume regions of the corneal epithelium or stroma. Potentially, it might also facilitate more reliable imaging of the endothelium.

## Conclusion

The present contribution has described for the first time a workflow to generate a volume representation of the entire imaged tissue region from laterally extended 3D-IVCM datasets acquired with oscillating focus scans. We have proposed three novel techniques for volume reconstruction with different degrees of computational complexity and evaluated them on 15 datasets of the SNP and the immediately adjacent tissue layers, from the posterior part of the epithelium to the anterior parts of the stroma. The presented results from 15 datasets acquired from 15 persons diagnosed with different forms of dry eye disease suggest that for the examined use case, determining the depth coordinate of the image data directly from the logged depth coordinates of the focus drive is sufficient for a visually adequate volume reconstruction. However, an additional subsequent correction step for the associated depth coordinates does improve the quality of the resulting SNP volume representation, which may be relevant for subsequent image analysis tasks. For different use cases, particularly regarding non-contact imaging, axial position correction may even be necessary. The primary intended application of the described workflow is to capture multiple datasets of the SNP region with small time increments to be able to perform accurate examinations of the dynamic behavior of ICs over a wide lateral region, including the possibility to asses potential axial movement of the cells. The flexibility of the workflow makes other potential use cases possible as well, for example the examination of the epithelial surface, the endothelial layer, stromal nerves, or macrophages in different corneal tissues.

## Supplementary Information

Below is the link to the electronic supplementary material.

Supplementary Material 1

## Data Availability

The datasets used and/or analysed during the current study are available from the corresponding author on reasonable request.
