# Supplementary material for: Large-area 3D reconstruction of corneal tissues from oscillating focus confocal microscopy
Source: Sci Rep. 2026 Apr 17;16:12693. doi: 10.1038/s41598-026-48735-5 (PMC13090340; doi:10.1038/s41598-026-48735-5)
Supplement: Supplementary file 1 — Supplementary Material 1 [file 41598_2026_48735_MOESM1_ESM.docx]

Large-area 3D reconstruction of corneal tissues from oscillating focus confocal microscopy

Stephan Allgeier*^1^, Sebastian Bohn^2,3^, Ralf Mikut^1^, Klaus-Martin Reichert^1^, Oliver Stachs^2,3^, Elisabeth Wagner^2^, Uwe Klaus Zettl^4^, Karsten Sperlich^2,3^

* Corresponding author: Stephan Allgeier
stephan.allgeier@kit.edu
Karlsruhe Institute of Technology, Institute for Automation and Applied Informatics, P.O. Box 3640, 76021 Karlsruhe, Germany

^1^ Institute for Automation and Applied Informatics, Karlsruhe Institute of Technology (KIT), Karlsruhe, Germany

^2^ Department of Ophthalmology, Rostock University Medical Center, Rostock, Germany

^3^ Department Life, Light & Matter, University of Rostock, Rostock, Germany

^4^ Neuroimmunology Section, Department of Neurology, Rostock University Medical Center, Rostock, Germany

Supplementary Figures: 16

Supplementary Figures


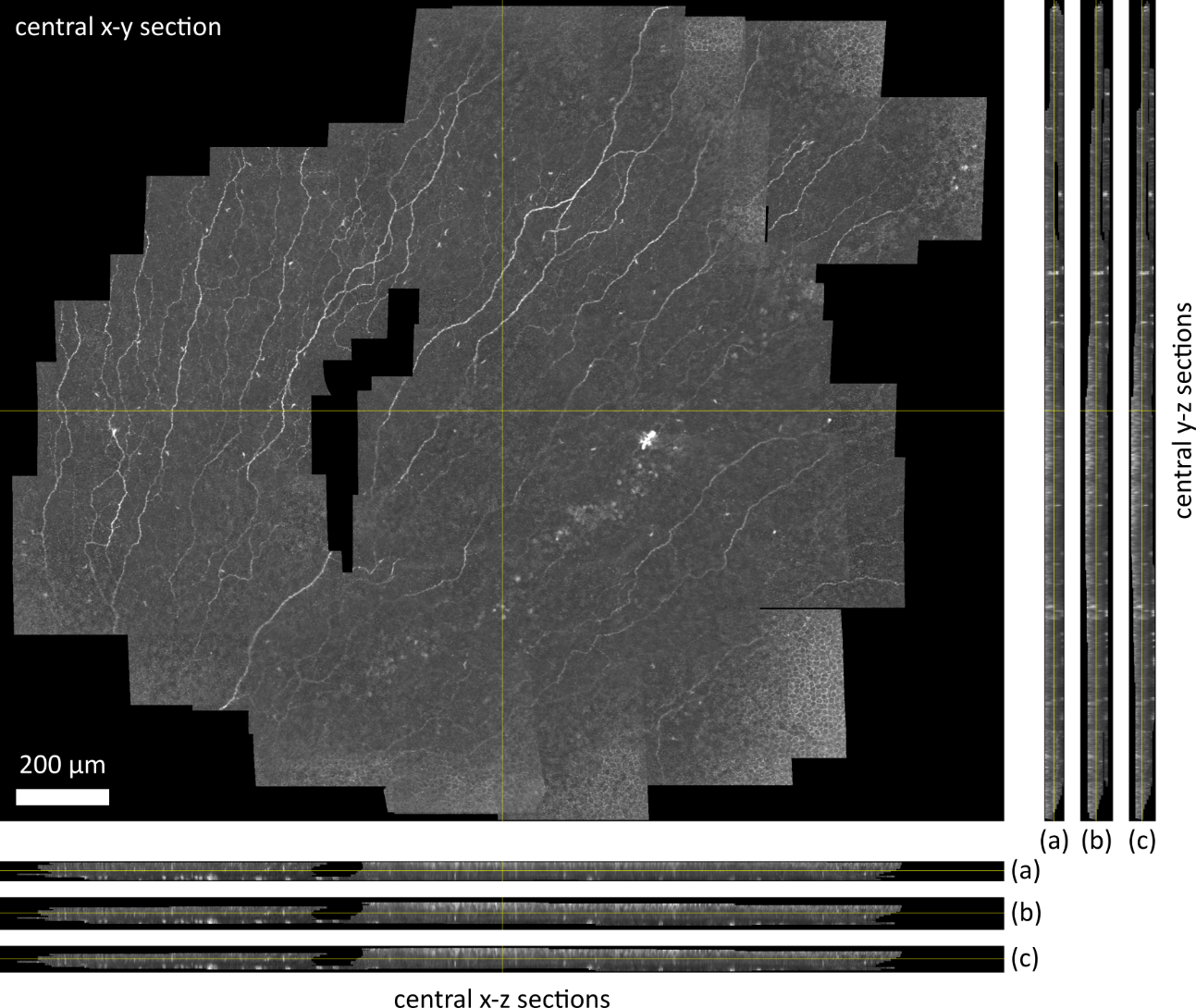


Supplementary Figure 1: Orthogonal sections through the reconstructed merged volume of dataset 1. The section planes are parallel to the x-y-plane (frontal, large image), the x-z-plane (sagittal, below the frontal image), and the y-z-plane (transversal, to the right of the frontal image). The transversal and sagittal sections from the results of methods (a), (b) and (c), as denoted next to the sections, have been taken at identical, central coordinates. The corneal surface is located near the top border of the sagittal section images and near the left border of the transversal section images.


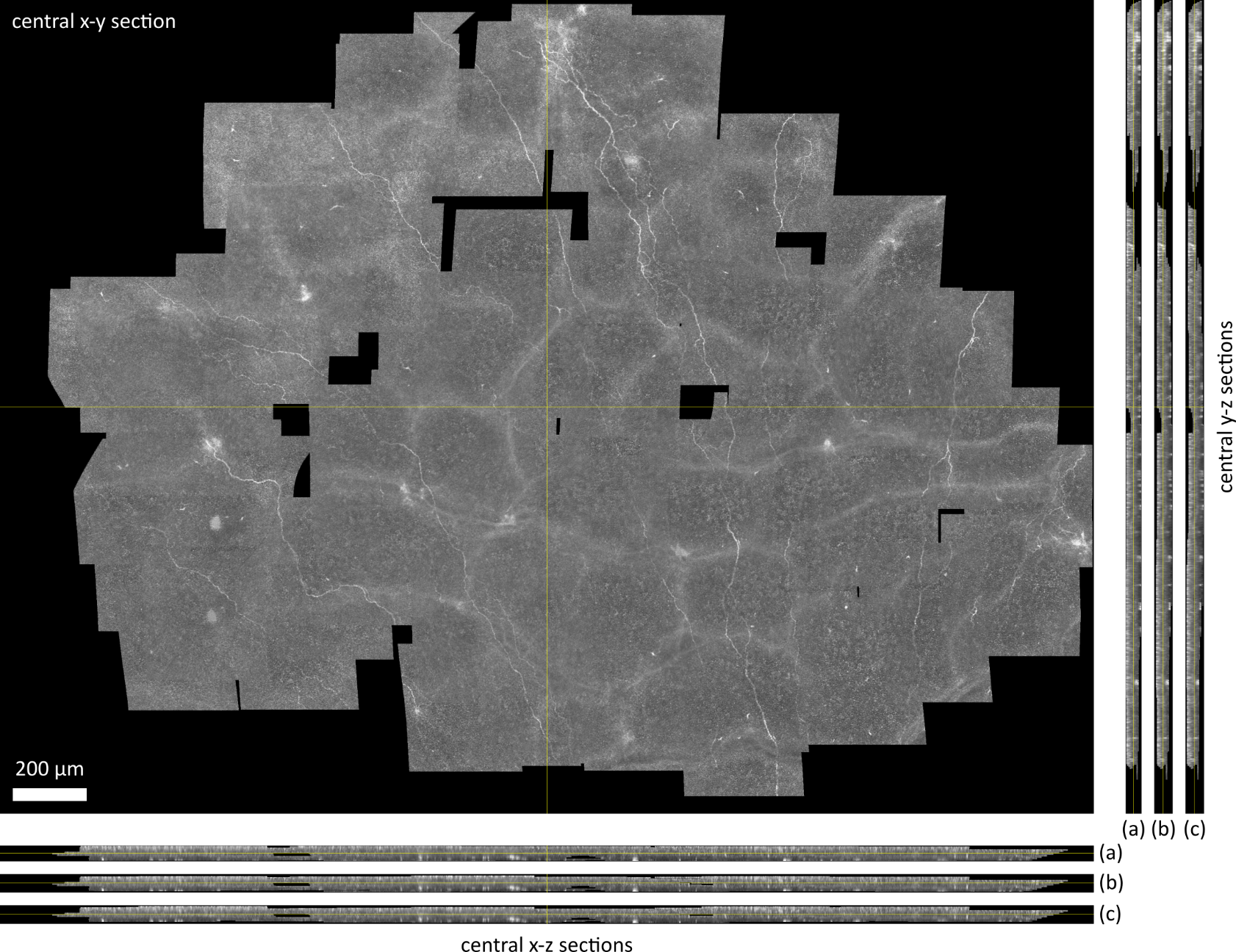


Supplementary Figure 2: Orthogonal sections through the reconstructed merged volume of dataset 2. The section planes are parallel to the x-y-plane (frontal, large image), the x-z-plane (sagittal, below the frontal image), and the y-z-plane (transversal, to the right of the frontal image). The transversal and sagittal sections from the results of methods (a), (b) and (c), as denoted next to the sections, have been taken at identical, central coordinates. The corneal surface is located near the top border of the sagittal section images and near the left border of the transversal section images.


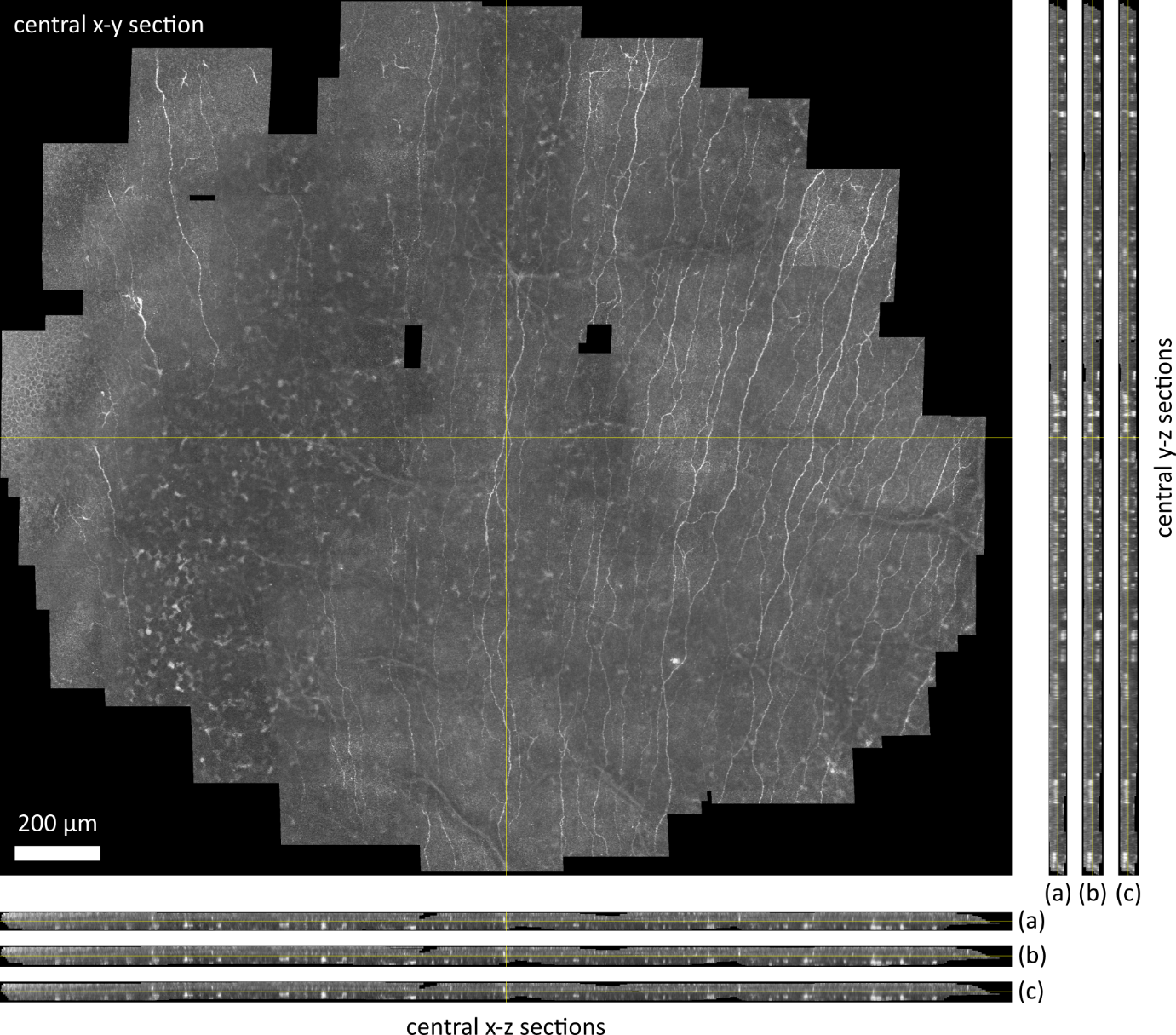


Supplementary Figure 3: Orthogonal sections through the reconstructed merged volume of dataset 3. The section planes are parallel to the x-y-plane (frontal, large image), the x-z-plane (sagittal, below the frontal image), and the y-z-plane (transversal, to the right of the frontal image). The transversal and sagittal sections from the results of methods (a), (b) and (c), as denoted next to the sections, have been taken at identical, central coordinates. The corneal surface is located near the top border of the sagittal section images and near the left border of the transversal section images.


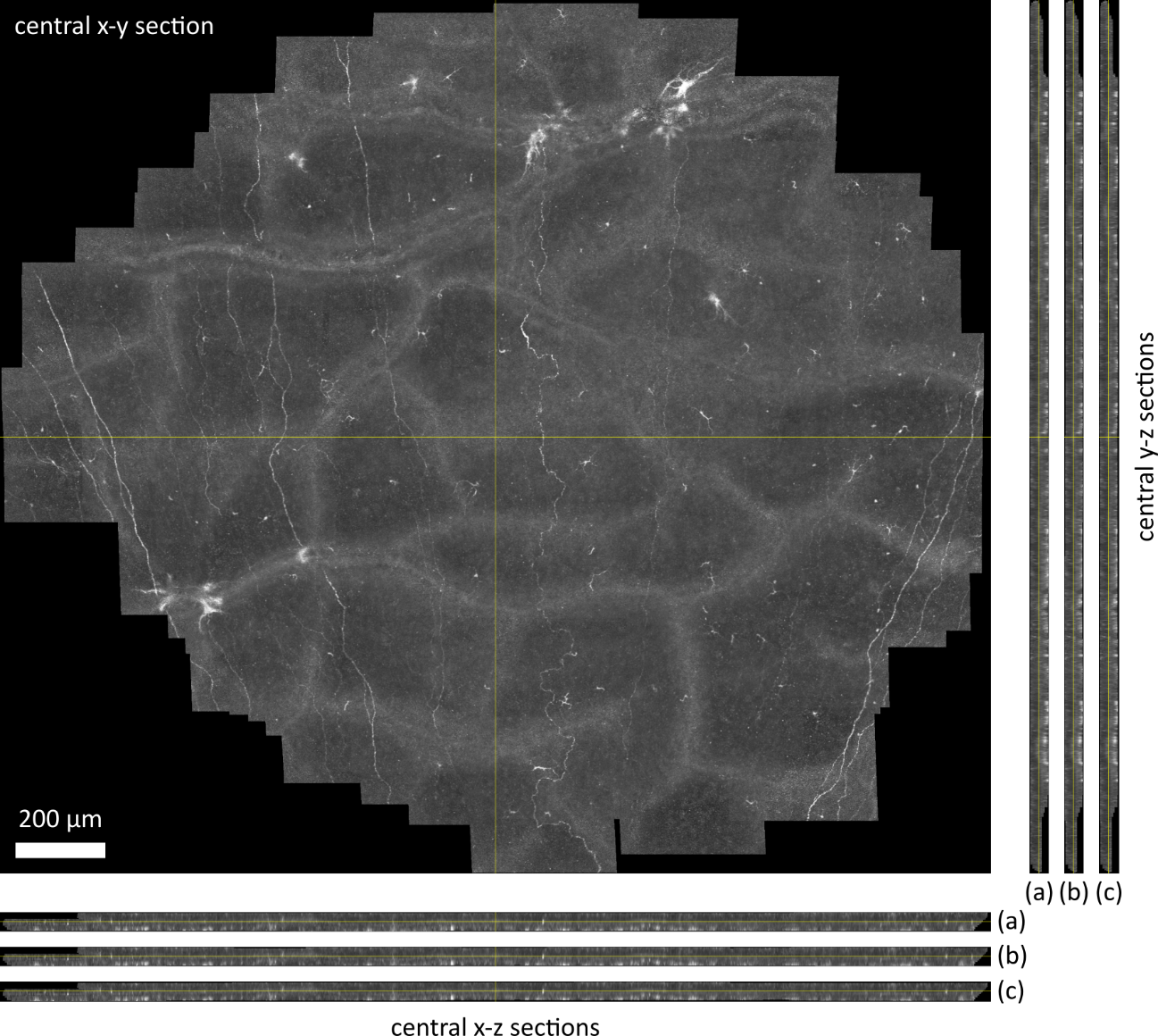


Supplementary Figure 4: Orthogonal sections through the reconstructed merged volume of dataset 4. The section planes are parallel to the x-y-plane (frontal, large image), the x-z-plane (sagittal, below the frontal image), and the y-z-plane (transversal, to the right of the frontal image). The transversal and sagittal sections from the results of methods (a), (b) and (c), as denoted next to the sections, have been taken at identical, central coordinates. The corneal surface is located near the top border of the sagittal section images and near the left border of the transversal section images.


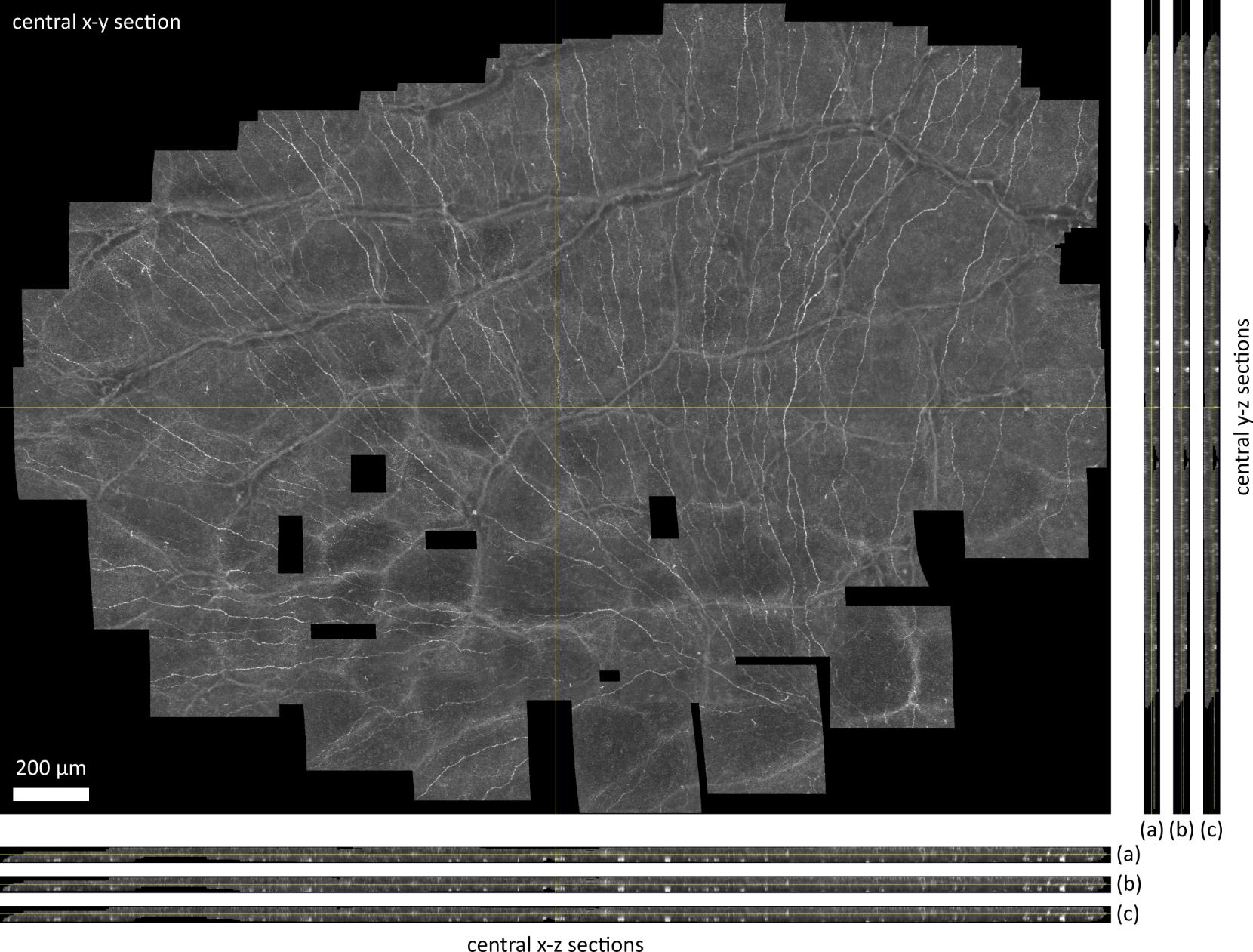


Supplementary Figure 5: Orthogonal sections through the reconstructed merged volume of dataset 5. The section planes are parallel to the x-y-plane (frontal, large image), the x-z-plane (sagittal, below the frontal image), and the y-z-plane (transversal, to the right of the frontal image). The transversal and sagittal sections from the results of methods (a), (b) and (c), as denoted next to the sections, have been taken at identical, central coordinates. The corneal surface is located near the top border of the sagittal section images and near the left border of the transversal section images.


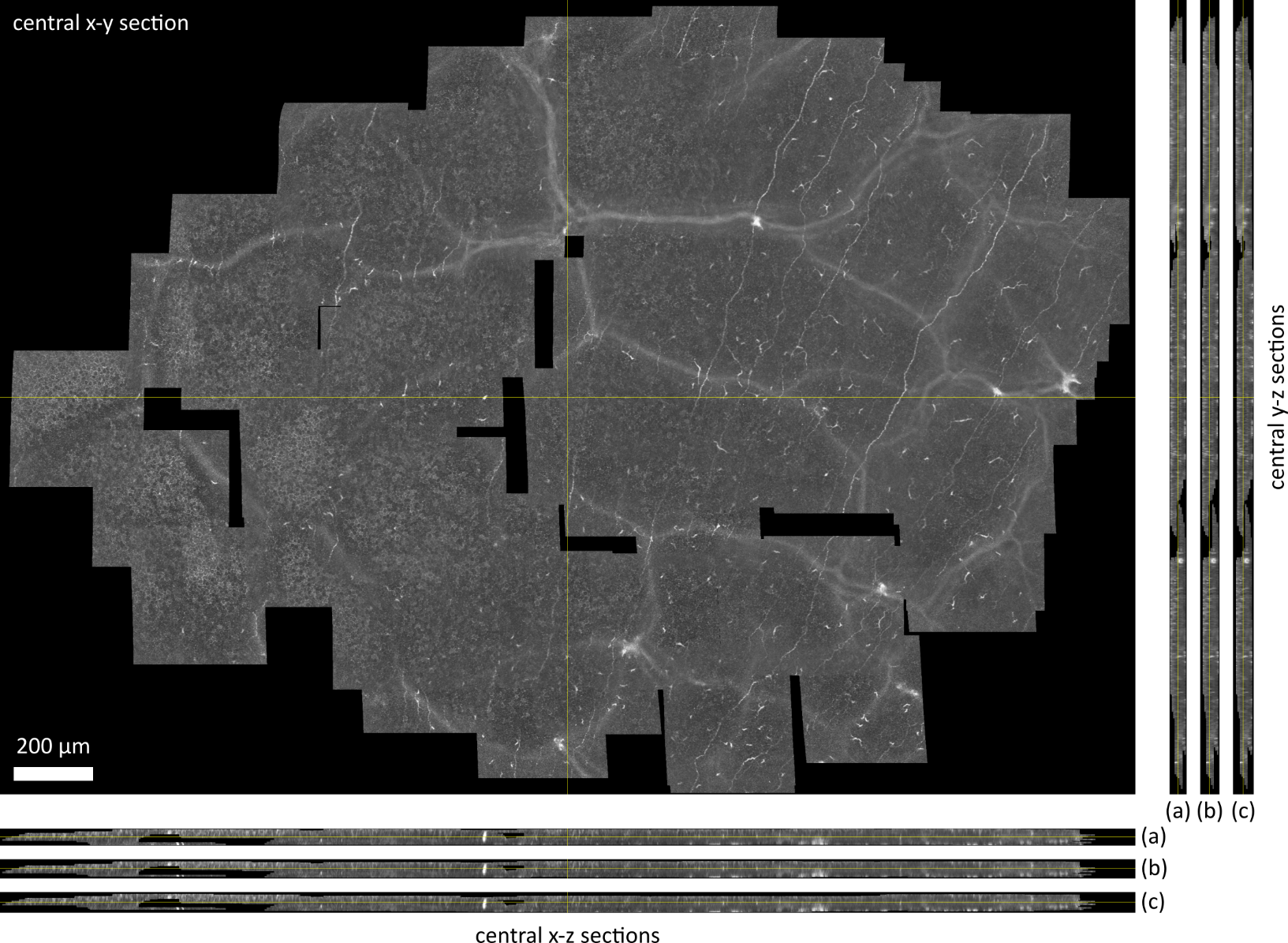


Supplementary Figure 6: Orthogonal sections through the reconstructed merged volume of dataset 6. The section planes are parallel to the x-y-plane (frontal, large image), the x-z-plane (sagittal, below the frontal image), and the y-z-plane (transversal, to the right of the frontal image). The transversal and sagittal sections from the results of methods (a), (b) and (c), as denoted next to the sections, have been taken at identical, central coordinates. The corneal surface is located near the top border of the sagittal section images and near the left border of the transversal section images.


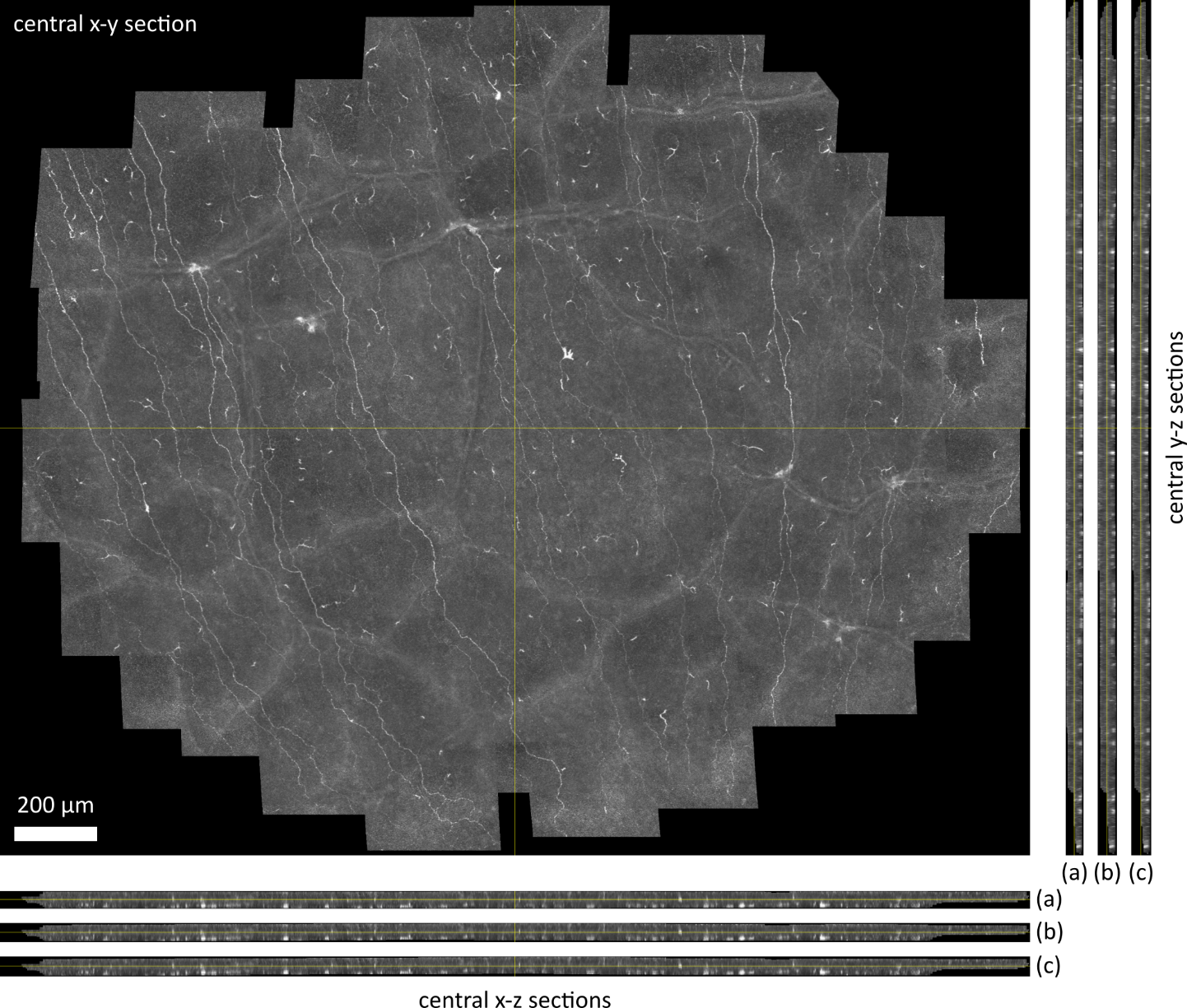


Supplementary Figure 7: Orthogonal sections through the reconstructed merged volume of dataset 7. The section planes are parallel to the x-y-plane (frontal, large image), the x-z-plane (sagittal, below the frontal image), and the y-z-plane (transversal, to the right of the frontal image). The transversal and sagittal sections from the results of methods (a), (b) and (c), as denoted next to the sections, have been taken at identical, central coordinates. The corneal surface is located near the top border of the sagittal section images and near the left border of the transversal section images.


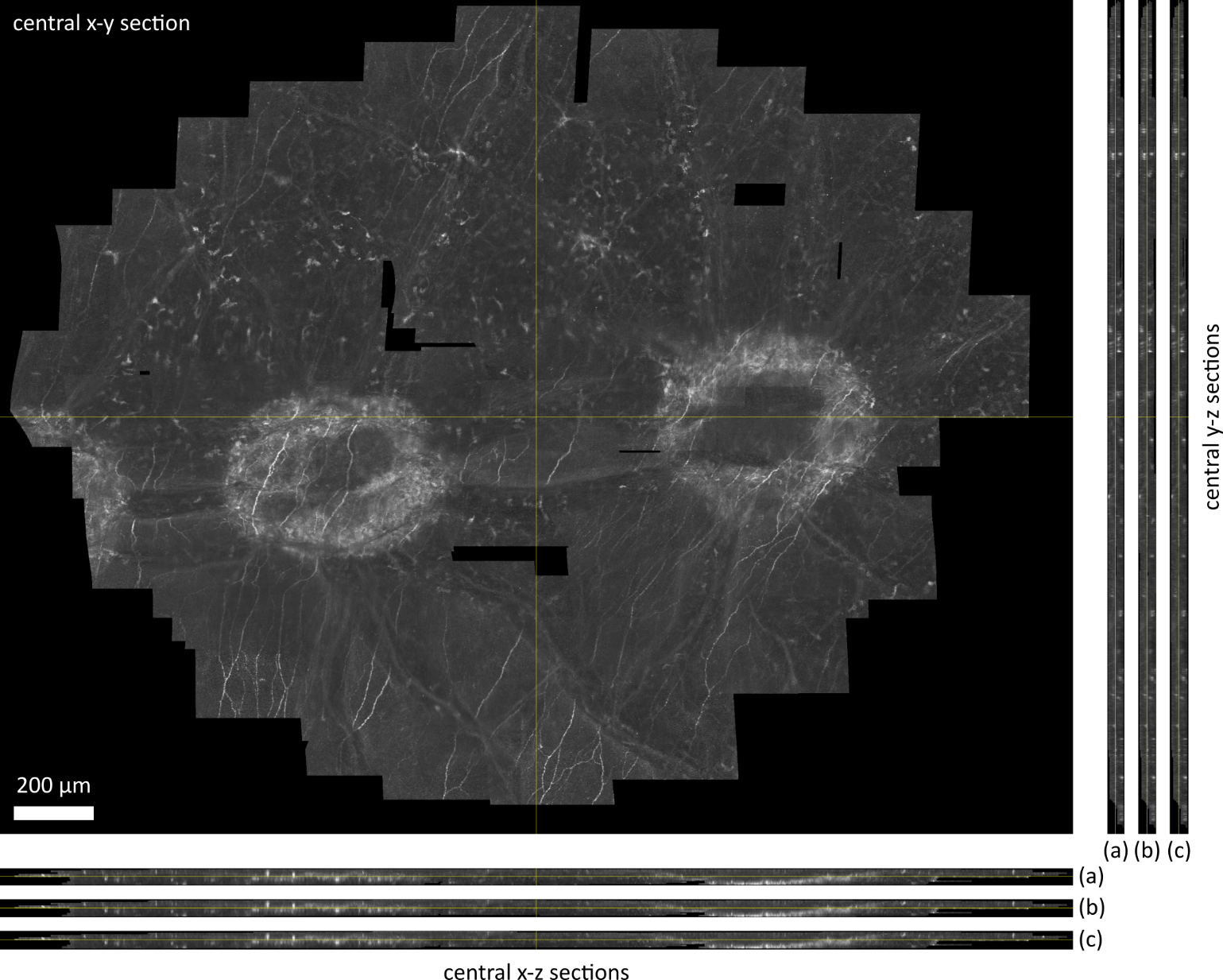


Supplementary Figure 8: Orthogonal sections through the reconstructed merged volume of dataset 8. The section planes are parallel to the x-y-plane (frontal, large image), the x-z-plane (sagittal, below the frontal image), and the y-z-plane (transversal, to the right of the frontal image). The transversal and sagittal sections from the results of methods (a), (b) and (c), as denoted next to the sections, have been taken at identical, central coordinates. The corneal surface is located near the top border of the sagittal section images and near the left border of the transversal section images.


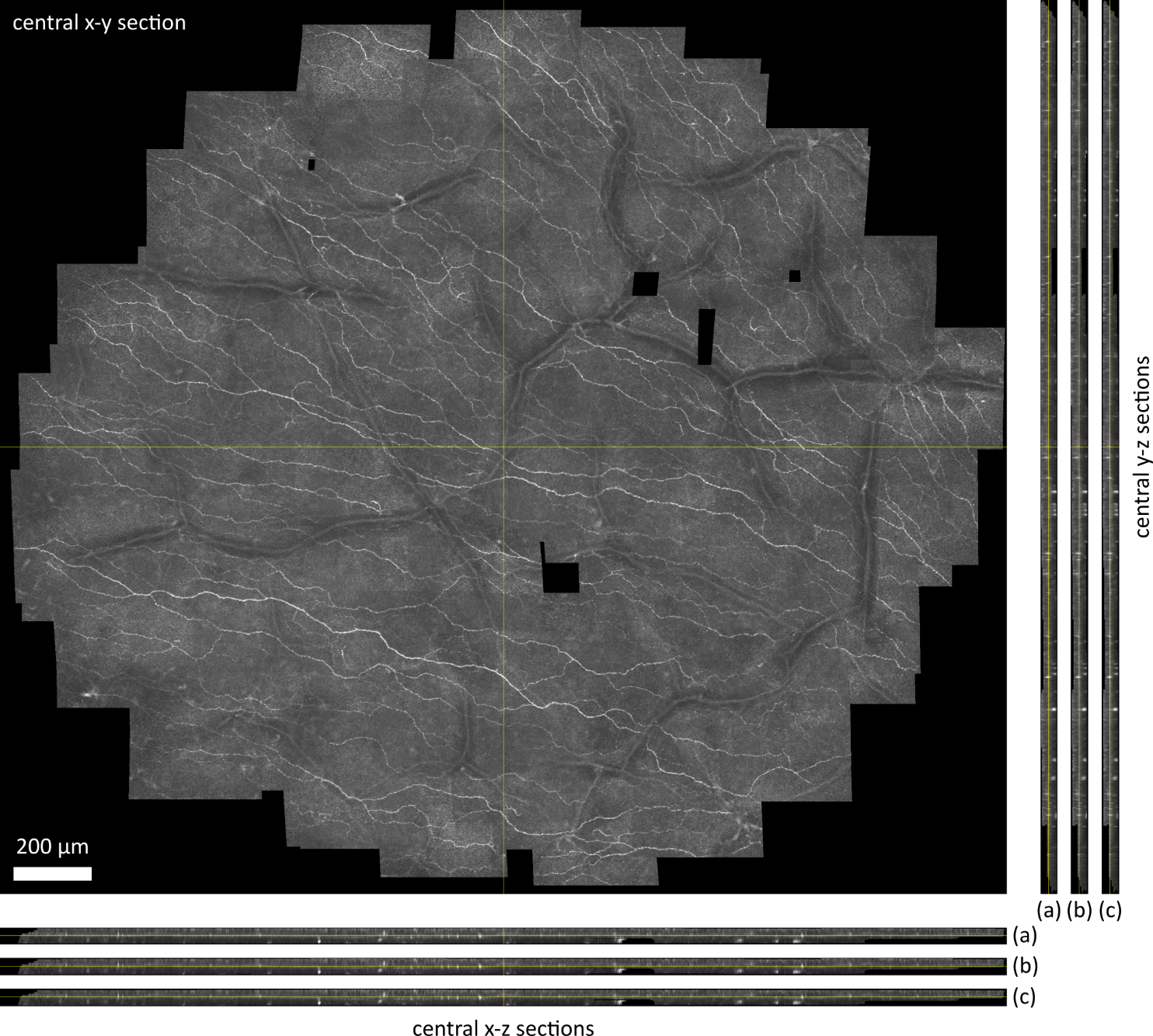


Supplementary Figure 9: Orthogonal sections through the reconstructed merged volume of dataset 9. The section planes are parallel to the x-y-plane (frontal, large image), the x-z-plane (sagittal, below the frontal image), and the y-z-plane (transversal, to the right of the frontal image). The transversal and sagittal sections from the results of methods (a), (b) and (c), as denoted next to the sections, have been taken at identical, central coordinates. The corneal surface is located near the top border of the sagittal section images and near the left border of the transversal section images.


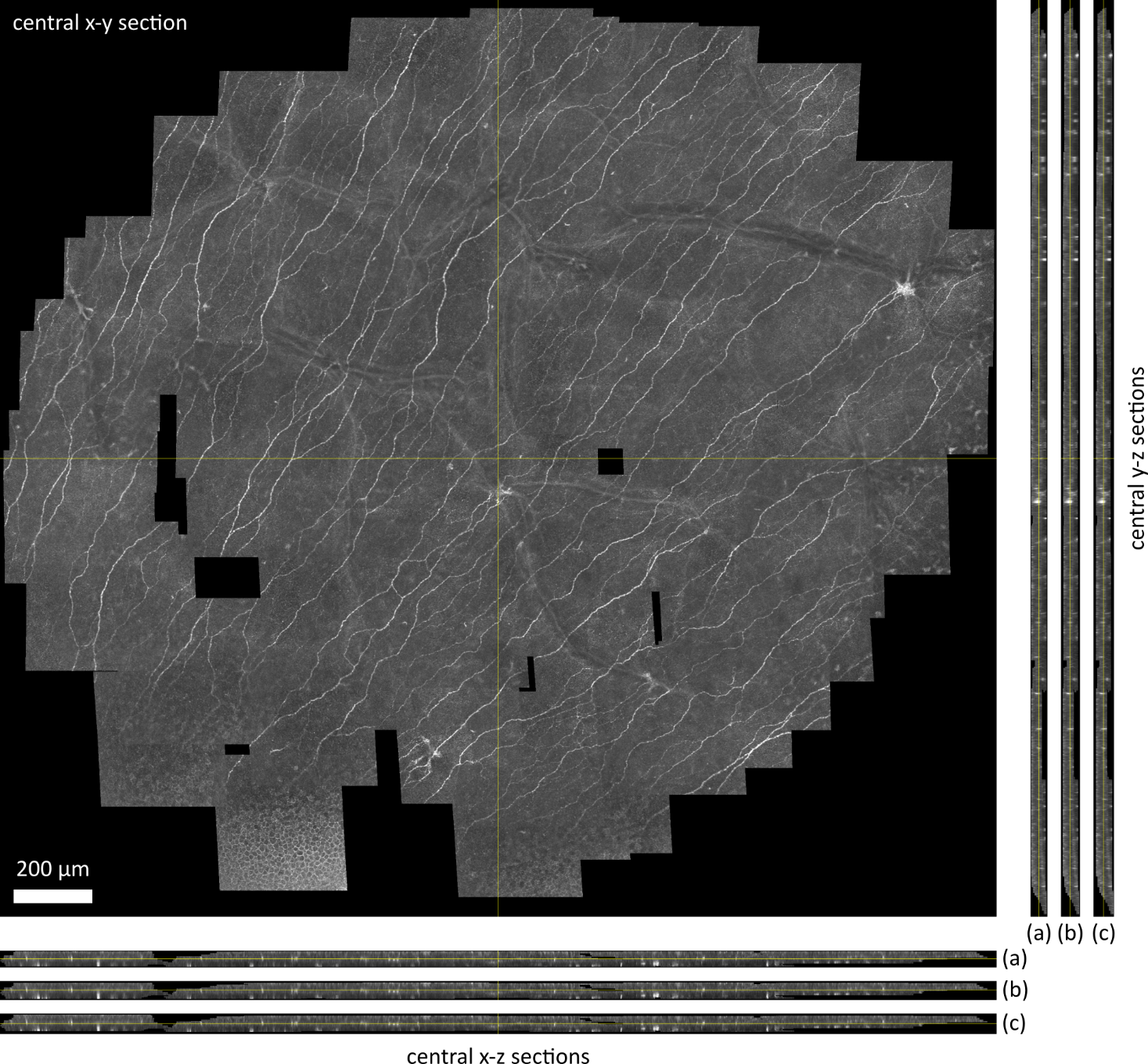


Supplementary Figure 10: Orthogonal sections through the reconstructed merged volume of dataset 10. The section planes are parallel to the x-y-plane (frontal, large image), the x-z-plane (sagittal, below the frontal image), and the y-z-plane (transversal, to the right of the frontal image). The transversal and sagittal sections from the results of methods (a), (b) and (c), as denoted next to the sections, have been taken at identical, central coordinates. The corneal surface is located near the top border of the sagittal section images and near the left border of the transversal section images.


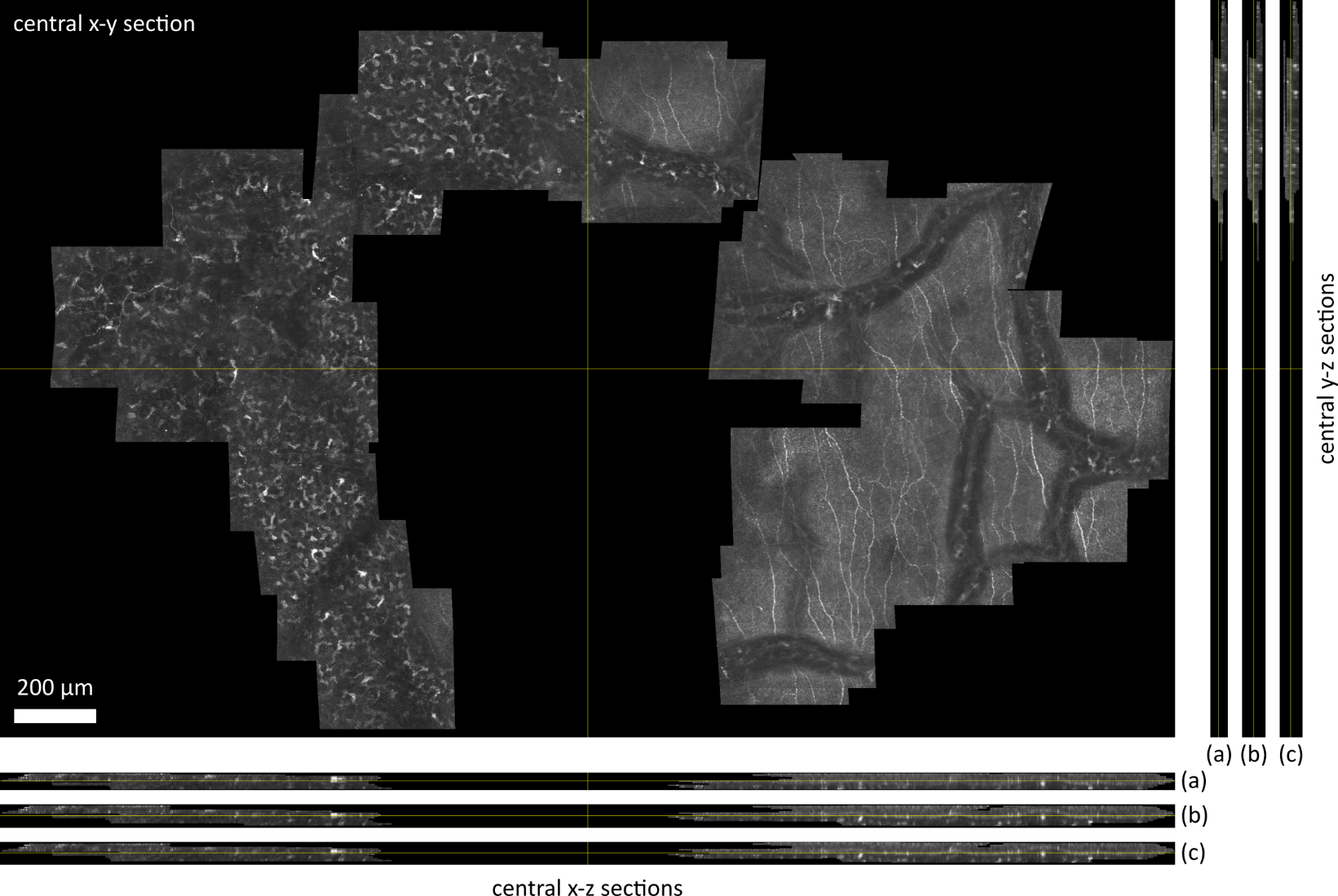


Supplementary Figure 11: Orthogonal sections through the reconstructed merged volume of dataset 11. The section planes are parallel to the x-y-plane (frontal, large image), the x-z-plane (sagittal, below the frontal image), and the y-z-plane (transversal, to the right of the frontal image). The transversal and sagittal sections from the results of methods (a), (b) and (c), as denoted next to the sections, have been taken at identical, central coordinates. The corneal surface is located near the top border of the sagittal section images and near the left border of the transversal section images. (The missing data in the central region is present in the raw data of this dataset but was excluded by the lateral motion correction and alignment step. This data exclusion can be avoided in principle by adjusting the process parameters for this individual dataset, after visual inspection of the results. The authors made the deliberate decision not to use dataset-specific process parameters, but instead use the exact same parameter settings for all 15 datasets.)


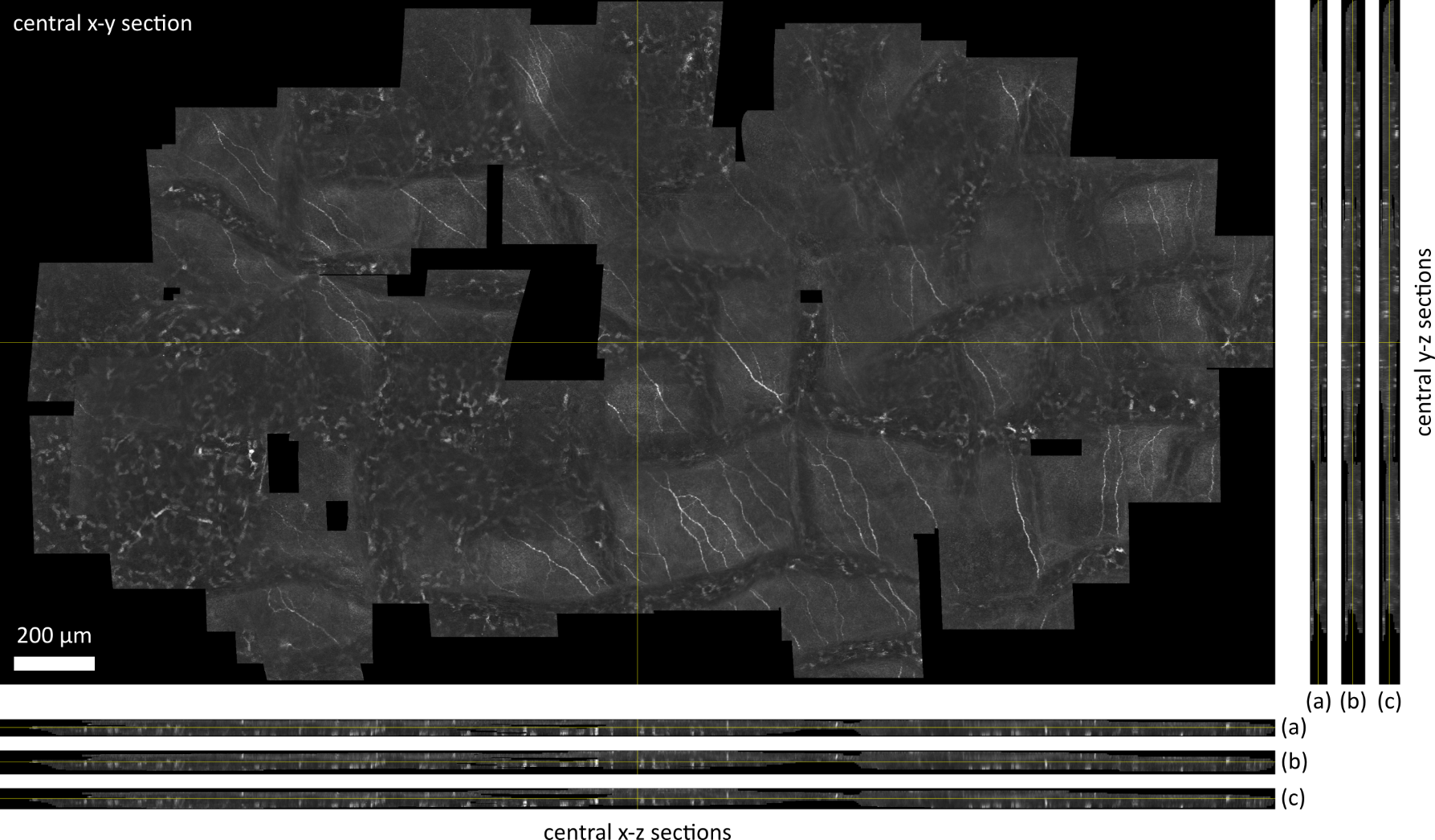


Supplementary Figure 12: Orthogonal sections through the reconstructed merged volume of dataset 12. The section planes are parallel to the x-y-plane (frontal, large image), the x-z-plane (sagittal, below the frontal image), and the y-z-plane (transversal, to the right of the frontal image). The transversal and sagittal sections from the results of methods (a), (b) and (c), as denoted next to the sections, have been taken at identical, central coordinates. The corneal surface is located near the top border of the sagittal section images and near the left border of the transversal section images.


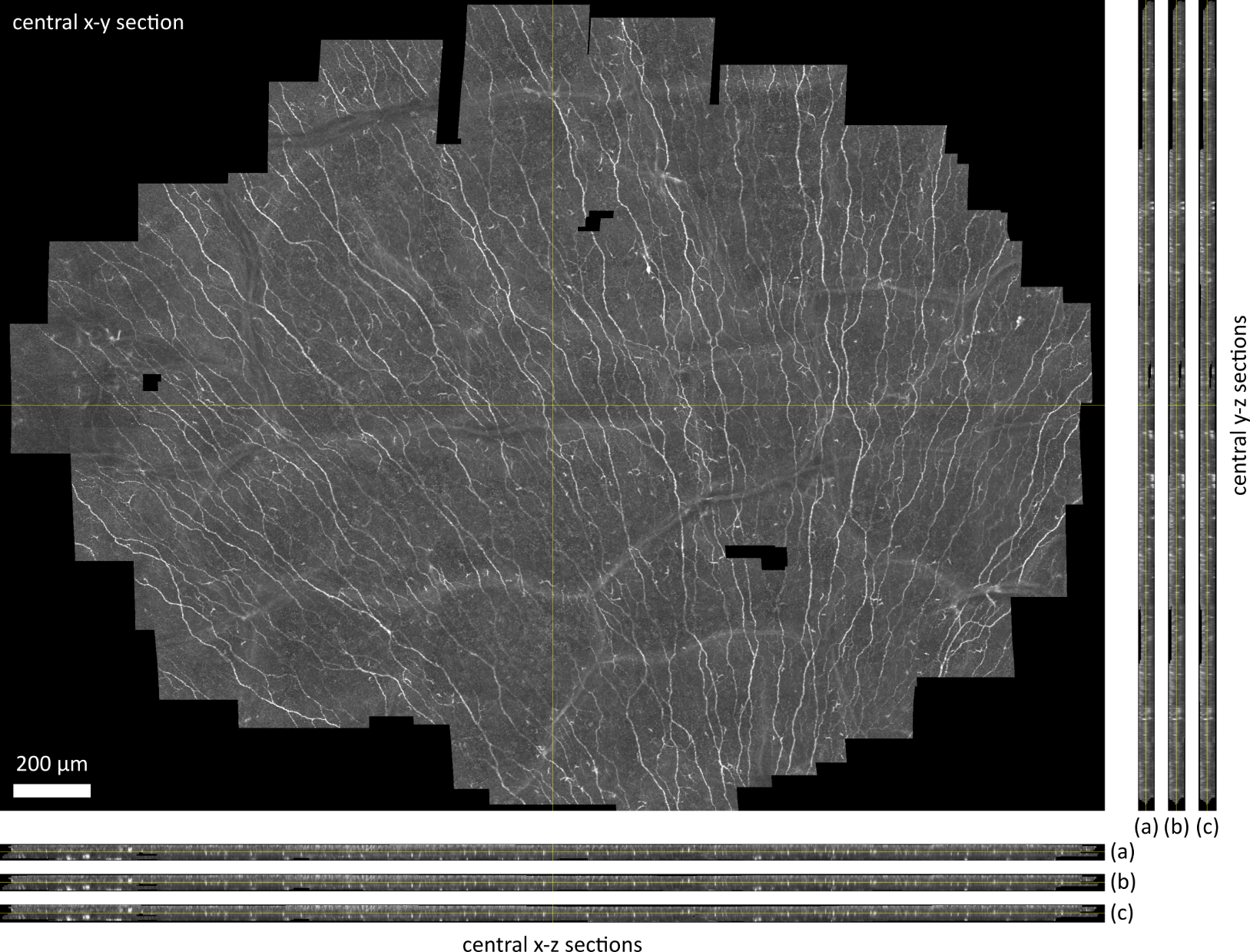


Supplementary Figure 13: Orthogonal sections through the reconstructed merged volume of dataset 13. The section planes are parallel to the x-y-plane (frontal, large image), the x-z-plane (sagittal, below the frontal image), and the y-z-plane (transversal, to the right of the frontal image). The transversal and sagittal sections from the results of methods (a), (b) and (c), as denoted next to the sections, have been taken at identical, central coordinates. The corneal surface is located near the top border of the sagittal section images and near the left border of the transversal section images.


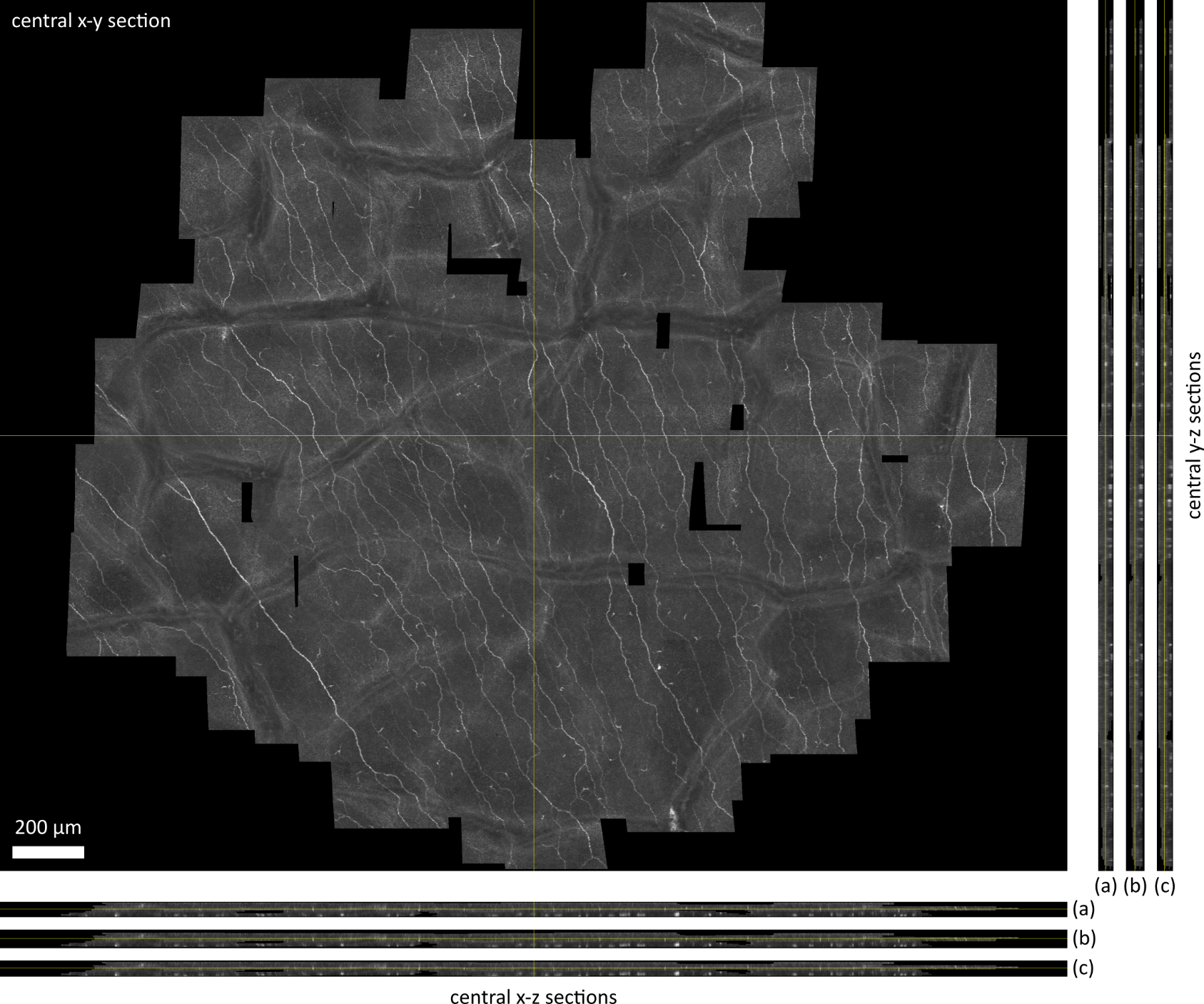


Supplementary Figure 14: Orthogonal sections through the reconstructed merged volume of dataset 14. The section planes are parallel to the x-y-plane (frontal, large image), the x-z-plane (sagittal, below the frontal image), and the y-z-plane (transversal, to the right of the frontal image). The transversal and sagittal sections from the results of methods (a), (b) and (c), as denoted next to the sections, have been taken at identical, central coordinates. The corneal surface is located near the top border of the sagittal section images and near the left border of the transversal section images.


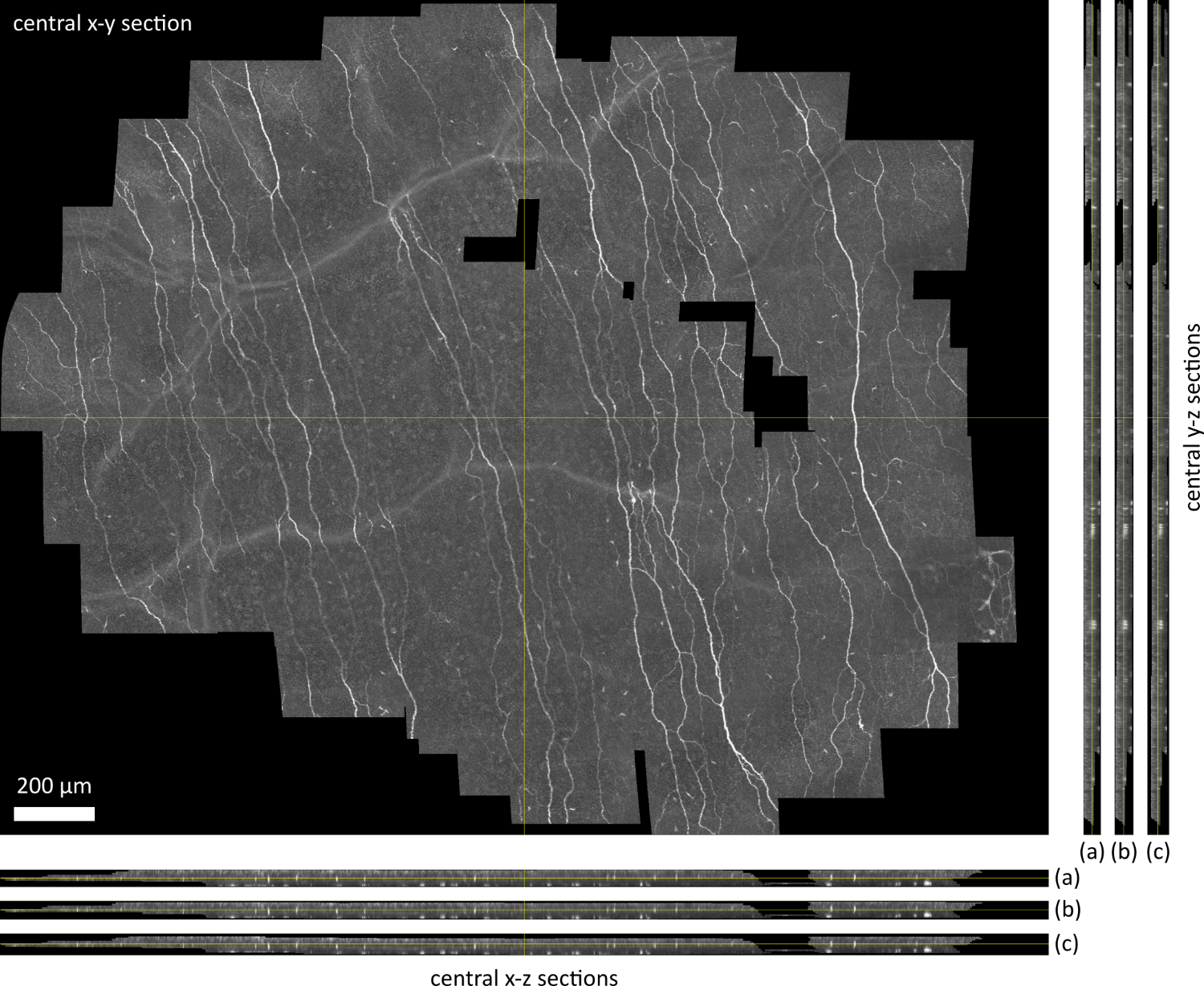


Supplementary Figure 15: Orthogonal sections through the reconstructed merged volume of dataset 15. The section planes are parallel to the x-y-plane (frontal, large image), the x-z-plane (sagittal, below the frontal image), and the y-z-plane (transversal, to the right of the frontal image). The transversal and sagittal sections from the results of methods (a), (b) and (c), as denoted next to the sections, have been taken at identical, central coordinates. The corneal surface is located near the top border of the sagittal section images and near the left border of the transversal section images.


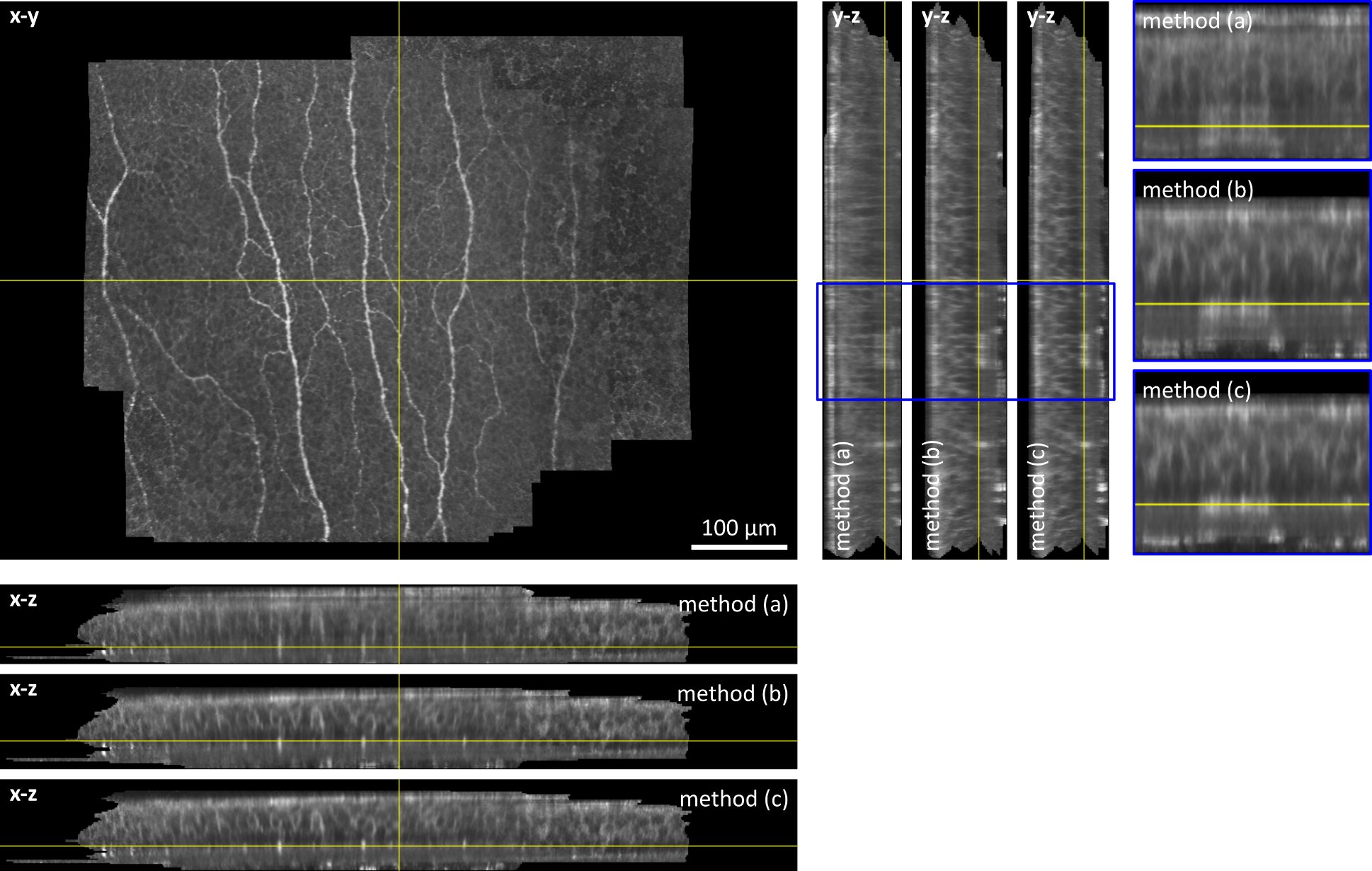


Supplementary Figure S16: Orthogonal sections through the reconstructed merged volume of an example dataset with several focus oscillations through the entire depth of the epithelium of a participant. The imaging procedure differs from the SNP imaging procedure by a larger focus oscillation amplitude of 80 µm, and the absence of voluntary eye movements. Instead, the participant was asked to fixate a static mark shown on the EyeGuidance display. The section planes are parallel to the x-y-plane (frontal, large image), the x-z-plane (sagittal, below the frontal image), and the y-z-plane (transversal, to the right of the frontal image), as denoted in the images. The transversal and sagittal sections from the results of methods (a), (b) and (c), as denoted in the images, have been taken at the identical, central coordinates. The corneal surface is visible as a horizontal layer that is significantly brighter than the adjacent epithelial tissue. The corneal surface is located near the top border of the sagittal section images and near the left border of the transversal section images. The region outlined with a blue rectangle in the three transversal section images is shown magnified (and rotated by 90 degrees) to the right of the transversal section images. The magnified region reveals a visually improved representation of the cellular structure with methods (b) and (c) over method (a). The reflective structures at the anterior end of the depicted tissue (at the bottom of the magnified section images) further reveal further superiority of method (c) over method (b), as the method (c) result shows better registration of these structures than the method (b) result.
